# Efficacy of antiresorptive agents bisphosphonates and denosumab in mitigating hypercalcemia and bone loss in primary hyperparathyroidism: A systematic review and meta-analysis

**DOI:** 10.3389/fendo.2023.1098841

**Published:** 2023-02-02

**Authors:** Swati Rajput, Aditya Dutta, Singh Rajender, Ambrish Mithal, Naibedya Chattopadhyay

**Affiliations:** ^1^ Division of Endocrinology and Centre for Research in Anabolic Skeletal Targets in Health and Illness (ASTHI), CSIR-Central Drug Research Institute, Lucknow, India; ^2^ Academy of Scientific and Innovative Research (AcSIR), Ghaziabad, India; ^3^ Institute of Endocrinology and Diabetes, Max Healthcare, Institutional Area, Press Enclave Road, Saket, New Delhi, India

**Keywords:** primary hyperparathyroidism, bisphosphonates, denosumab, bone mineral density, bone turnover markers, anti-resorptives

## Abstract

**Purpose:**

Primary hyperparathyroidism (PHPT) is characterized by increased bone remodeling and hypercalcemia. Parathyroidectomy (PTX), the current standard of care, is recommended in all symptomatic and some groups of asymptomatic patients. Anti-resorptive therapies (bisphosphonates and denosumab) have been used in patients where PTX is refused or contraindicated. In this meta-analysis, we investigated the effectiveness of anti-resorptives in preventing/treating PHPT-induced bone loss and mitigating hypercalcemia.

**Method:**

PubMed, Scopus, and Cochrane Library databases were searched for articles with keywords containing PHPT, bisphosphonates, and denosumab in various combinations. We extracted and tabulated areal BMD (aBMD), serum mineral, and bone turnover parameters from the qualified studies and used comprehensive meta-analysis software for analysis.

**Results:**

Of the 1,914 articles screened, 13 were eligible for meta-analysis. In the pooled analysis, 12 months of anti-resoptives (bisphosphonates and denosumab) therapy significantly increased aBMD at the lumbar spine (Standard difference in means (SDM)=0.447, 95% CI=0.230 to 0.664, p=0.0001), femoral neck (SDM=0.270, 95% CI=0.049 to 0.491, p=0.017) and increased serum PTH (SDM=0.489, 95% CI=0.139 to 0.839, p=0.006), and decreased serum calcium (SDM=-0.545, 95% CI=-0.937 to -0.154, p=0.006) compared with baseline. 12 months of bisphosphonate use significantly increased aBMD only at the lumbar spine (SDM=0.330, 95% CI=0.088 to 0.571, p=0.007) with a significant increased in serum PTH levels (SDM=0.546, 95% CI= 0.162 to 0.930, p=0.005), and a decreased in serum calcium (SDM=-0.608, 95% CI=-1.048 to -0.169, p=0.007) and bone-turnover markers (BTMs) compared with baseline. Denosumab use for 12 months significantly increased aBMD at both the lumbar spine (SDM=0.828, 95% CI=0.378 to 1.278, p=0.0001) and femur neck (SDM=0.575, 95% CI=0.135 to 1.015, p=0.010) compared with baseline. Mean lumbar spine aBMD (SDM=0.350, 95% CI=0.041 to 0.659, p=0.027) and serum PTH (SDM=0.602, 95% CI= 0.145 to 1.059, p=0.010) were significantly increased after 12 months of alendronate use compared with placebo. When compared with baseline, alendronate significantly decreased BTMs after 12 months and increased aBMD without altering the PTH and calcium levels after 24 months.

**Conclusion:**

Anti-resorptives are effective in mitigating bone loss and hypercalcemia in PHPT while maintaining or increasing aBMD. PTX reversed all changes in PHPT and normalized PTH levels.

## 1 Introduction

Primary hyperparathyroidism (PHPT) is a disorder of mineral metabolism that is commonly observed in women of age 50 to 60 years (1–3). It is characterized by autonomous parathyroid hormone (PTH) secretion resulting in myriad systemic manifestations such as bone mineral loss, osteoporosis, fractures, lytic lesions, renal stones, and hypercalcemia (3). Low bone mineral density (BMD), osteopenia, and osteoporosis are frequently observed in women with PHPT (4). PHPT is characterized by an increase in the activation frequency of bone multicellular units (BMUs), resulting in an enlarged bone remodeling space. Specifically, cortical bone porosity and endocortical bone resorption are increased, leading to cortical bone loss with relative preservation of trabecular bones (5). These skeletal events account for increased calcium and bone turnover markers (BTM) in both serum and urine (6). PHPT increases fracture risk; thus, treatment strategies aiming to ameliorate hypercalcemia and improving BMD are likely to be clinically relevant.

Parathyroidectomy (PTX) is the standard of care for treating symptomatic PHPT and, in some cases of asymptomatic PHPT (3). According to the guidelines issued by the Third International Workshop on the Management of Asymptomatic Primary Hyperparathyroidism, PTX has been recommended for those with osteoporosis (T-score ≤ −2.5 at the hip, spine, or one-third distal radial site), hypercalcemia (serum calcium > 0.25 mmol/L above normal), creatinine clearance below 60 mL/min, or age < 50 years (7). Besides restoring normocalcemia, PTX increases BMD and decreases fracture risk in patients with osteoporosis and osteopenia (8). However, up to 75-80% of PHPT patients are asymptomatic at the time of presentation (9), and not everyone fits the aforementioned criteria for surgery. Therefore, specific pharmacotherapy targeting hypercalcemia and/or low BMD may be beneficial if the patient does not meet surgical requirements or presents with some medical contraindication/is unwilling for surgery (7).

Current pharmacotherapy for PHPT consists of calcimimetics (cinacalcet) to suppress PTH secretion (10) and anti-resorptive drugs, including bisphosphonates (BPs) and denosumab (RANKL neutralizing antibody) (11). Anti-resorptives are attractive because they increase BMD and reduce fracture risk in postmenopausal and senile osteoporosis patients. Because the characteristics of bone loss in PHPT differ from those seen in postmenopausal osteoporosis, it is essential to establish the usefulness of these therapies in increasing bone mass in PHPT. A systematic review observed that BPs improved BMD in PHPT patients but lowered serum calcium transiently (12).

This meta-analysis was undertaken to determine the effect of anti-resorptives (BPs, and/or denosumab) on areal BMD (aBMD), bone turnover markers (BTMs), calcium and phosphate levels in patients with PHPT (asymptomatic or surgery contraindicated) compared with placebo or baseline.

## 2 Method

### 2.1 Search strategy

**Figure 1:**
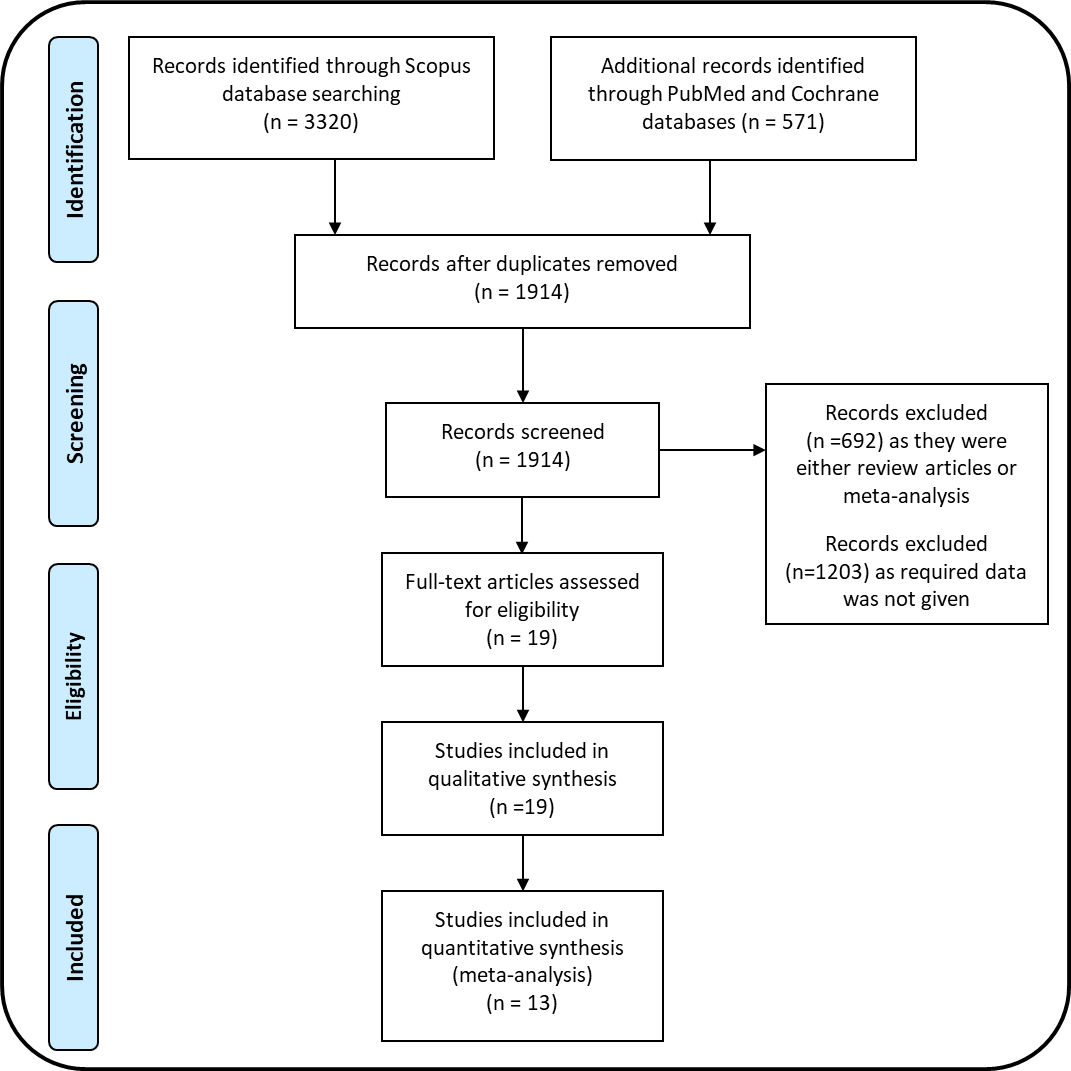
PRISMA flow diagram.

The electronic databases PubMed (1976 to May 2022), Scopus (1998 to May 2022), and Cochrane Library (until May 2022) were searched to identify the studies that assessed the effect of BPs, denosumab, and BPs or denosumab compared with PTX in PHPT patients. The search strategy included various combinations of keywords and Boolean operators. The search terms included “PHPT, bone, BPs”, “PHPT, bone, Denosumab”, “PHPT, bone, alendronate”, “PHPT, bone, zoledronate”, “PHPT, bone, risedronate”, “PHPT, bone, etidronate”, “PHPT, bone, ibandronate”, “PHPT, bone, clodronate”, “PHPT, bone, neridronate”. The PRISMA flow diagram shows the findings of literature search and screening of the studies (**Figure 1**).

### 2.2 Inclusion and exclusion criteria

Inclusion criteria were as follows (1): original research and full-text articles published in the English language (2), studies where the PHPT is confirmed by hypercalcemia and elevated PTH levels (3), studies where the bone parameters such as aBMD at any site such as the lumbar spine, femoral neck, total hip or distal radius and BTMs were measured (4), retrospective studies, prospective studies and randomized controlled trials, and (5) sufficient quantitative data (mean± SD/SEM) is presented. The exclusion criteria included (1) studies where the PTX and drug treatment were given simultaneously (2), data represented in median±interquartile range (3), aBMD represented in terms of t-score, z-score because reference value is not given, which can be used to convert t-score to g/cm^2^ (4), reviews, case reports, book chapters, and letters to the editor, and (5) articles published in languages other than English. There were no limitations with regard to age and gender.

### 2.3 Data extraction

Two reviewers (SR and NC) independently assessed the studies for their eligibility. Disagreements with the eligibility of studies were resolved by discussion with all authors. Data were extracted from each article in the numeric form from tables and bar/XY plots using the WebPlotDigitilizer program (13). Data were tabulated from each eligible study for the following parameters: author name, year of publication, number of patients, aBMD at different sites such as the lumbar spine, femoral neck, total hip, and distal radius, serum PTH levels, serum calcium, serum phosphate, serum osteocalcin (OCN), serum bone alkaline phosphatase (BALP), serum CTX-I, age, and gender. Extracted data were transformed from % change in mean to absolute change in mean after treatment using (after treatment – before treatment)/before treatment * 100 = % change. Data extracted from all articles included in the meta-analysis showed in **Table 1**.

**Table 1:** Data extracted from all articles included in the meta-analysis.

Parameters	Duration of drug administration	Number of patients	Age (Mean±SD) years/ Gender	Baseline Mean (SD)	After drug administration Mean (SD)	Reference
PTH (pMol/L)	12 months	Placebo= 13 ALN= 13	Placebo=74±4/ F ALN=72±5/F	Placebo=15.50 (2.80) ALN=14.40 (5.20)	Placebo=13.49 (3.58) ALN=16.56 (6.24)	Rossini et al. (14)
		EHDP= 9 PTX= 13	EHDP=76.3±5.2/F PTX=76.8±10.1/F	EHDP=8.42 (0.97) PTX=10.15 (1.11)	EHDP=11.88 (1.11) PTX=1.82 (1.32)	Horiuchi et al. (15)
		Placebo= 18 ALN= 14	Placebo= 63.4± 2.02/ ALN= 69.6± 2.91/ F=27 & M=5	Placebo=10.74 (2.10) ALN= 10.37 (1.84)	Placebo=11.84 (1.52) ALN= 12.199 (2.01)	Parker et al. (16)
		Placebo= 20 ALN= 20	Placebo= 71.8± 8.8/F ALN= 68.2± 9.7/F	Placebo=24.20 (15.10) ALN=19.90 (12.40)	Placebo=24.29 (2.86) ALN= 24.28 (2.87)	Chow et al. (17)
		Placebo= 19 ALN= 18	Placebo=70.09±10.36/M=6, F=13 ALN=63.73±9.36/ M=3, F=15	Placebo=15.43 (1.41) ALN=17.31 (4.20)	Placebo=15.51 (2.57) ALN=21.12 (6.16)	Khan et al. (18)
		Neridronate IV= 54	Neridronate IV=64±8/F	Neridronate IV=16.60 (11)	Neridronate IV=18.04 (11.69)	Rossini et al. (14)
		Placebo= 10 ALN= 12	Placebo=63.2±8.3/F ALN=69.4±6.3/F	Placebo=17.1 (6.50) ALN= 11.8 (3.35)	Placebo=18.1 (5.75) ALN=14 (4.20)	Akbaba et al. (19)
		ALN= 33 PTX= 33	F=60, M=3 54 post-menopausal, 6 pre-menopausal	ALN=14.98 (6.24) PTX=23.89 (21.66)	ALN=16.36 (6.84) PTX=4.65 (2.40)	Szymczak et al. (20)
		Placebo= 15 ALN= 15	Placebo=57±5/F ALN=59±5/F	Placebo=10.60 (1.30) ALN=11.20 (2.10)	Placebo=11 (1.40) ALN=11 (1.30)	Cesareo et al. (19)
		Placebo= 15 Denosumab= 16	Placebo=68·0 ±1·8/ M=3, F=12 Denosumab= 65·4±2·2/ M=3, F=13	Placebo=11.16 (6.66) Denosumab=12.95 (9.92)	Placebo=10.97 (9.33) Denosumab=13.51 (8.00)	Leere et al. (21)
		PTX= 24	PTX= 61.4±9.8/ M=7, F=17	PTX= 24.37 (20.39)	PTX= 4.37 (3.34)	Choe et al. (22)
	24 months	Placebo= 13 ALN= 13	Placebo=74±4/ F ALN=72±5/F	Placebo=15.50 (2.80) ALN= 14.40 (5.20)	Placebo=16.34 (3.28) ALN= 16.27 (6.71)	Rossini et al. (23)
		Placebo= 18 ALN= 14 After 24 months Placebo= 13 ALN= 10	Placebo= 63.4± 2.02/ ALN= 69.6± 2.91/ F=27 & M=5	Placebo=10.74 (2.10) ALN=10.37 (1.84)	Placebo=12.45 (1.94) ALN=11.93 (1.69)	Parker et al. (16)
		Placebo= 19 ALN= 18	Placebo=70.09±10.36/M=6, F=13 ALN=63.73±9.36/ M=3, F=15	Placebo=15.43 (1.41) ALN=17.31 (4.20)	ALN=15.67 (3.12)	Khan et al. (18)
		Neridronate IV= 54	Neridronate IV=64±8/F	Neridronate IV=16.60 (11)	Neridronate IV=26.30 (11.98)	Rossini et al. (14)
Calcium (mMol/L)	12 months	Placebo= 13 ALN= 13	Placebo=74±4/ F ALN=72±5/F	Placebo= 2.73 (0.08) ALN= 2.75 (0.10)	Placebo= 2.70 (0.08) ALN= 2.70 (0.10)	Rossini et al. (23)
		EHDP= 9 PTX= 13	EHDP=76.3±5.2/F PTX=76.8±10.1/F	EHDP= 2.71 (0.13) PTX= 2.76 (0.18)	EHDP= 2.58 (0.13) PTX= 2.39 (0.13)	Horiuchi et al. (15)
		Placebo= 18 ALN= 14	Placebo= 63.4± 2.02/ ALN= 69.6± 2.91/ F=27 & M=5	Placebo= 2.82 (0.08) ALN= 2.84 (0.07)	Placebo= 2.78 (0.08) ALN=2.87 (0.07)	Parker et al. (16)
		Placebo= 20 ALN= 20	Placebo= 71.8± 8.8/F ALN= 68.2± 9.7/F	Placebo= 2.81 (0.16) ALN= 2.82 (0.18)	Placebo= 2.83 (0.04) ALN=2.75 (0.05)	Chow et al. (17)
		Placebo= 19 ALN= 18	Placebo=70.09±10.36/M=6, F=13 ALN=63.73±9.36/ M=3, F=15	Placebo= 2.64 (0.03) ALN= 2.68 (0.03)	Placebo= 2.67 (0.05) ALN= 2.64 (0.02)	Khan et al. (18)
		Neridronate IV = 54	Neridronate IV=64±8/F	Neridronate IV = 2.68 (0.15)	Neridronate IV = 2.64 (0.15)	Rossini et al. (14)
		Placebo= 10 ALN= 12	Placebo=63.2±8.3/F ALN=69.4±6.3/F	Placebo= 2.78 (0.13) ALN= 2.80 (0.14)	Placebo= 2.75 (0.16) ALN= 2.68 (0.11)	Akbaba et al. (19)
		Placebo= 15 Denosumab= 16	Placebo=68·0 ±1·8/ M=3, F=12 Denosumab= 65·4±2·2/ M=3, F=13	Placebo= 2.71 (0.15) Denosumab= 2.72 (0.36)	Placebo= 2.72 (0.12) Denosumab= 2.68 (0.24)	Leere et al. (21)
		PTX= 24	PTX= 61.4±9.8/ M=7, F=17	PTX= 2.80 (0.60)	PTX=2.25 (0.15)	Choe et al. (22)
	24 months	Placebo= 13 ALN= 13	Placebo=74±4/ F ALN=72±5/F	Placebo= 2.73 (0.08) ALN= 2.75 (0.10)	Placebo= 2.73 (0.08) ALN= 2.77 (0.10)	Rossini et al. (23)
		Placebo= 18 ALN= 14 After 24 months Placebo= 13 ALN= 10	Placebo= 63.4± 2.02/ ALN= 69.6± 2.91/ F=27 & M=5	Placebo= 2.82 (0.08) ALN= 2.84 (0.07)	Placebo= 2.69 (0.07) ALN=2.89 (0.03)	Parker et al. (16)
		Placebo= 19 ALN= 18	Placebo=70.09±10.36/M=6, F=13 ALN=63.73±9.36/ M=3, F=15	Placebo= 2.64 (0.03) ALN= 2.68 (0.03)	ALN= 2.62 (0.04)	Khan et al. (18)
		Neridronate IV = 54	Neridronate IV=64±8/F	Neridronate IV = 2.68 (0.15)	Neridronate IV = 2.68 (0.15)	Rossini et al. (14)
Phosphate (mg/dL)	12 months	Placebo= 13 ALN= 13	Placebo=74±4/ F ALN=72±5/F	Placebo= 2.60 (0.50) ALN= 2.90 (0.60)	Placebo= 2.69 (0.62) ALN= 2.91 (0.66)	Rossini et al. (23)
		Placebo= 18 ALN= 14	Placebo= 63.4± 2.02/ ALN= 69.6± 2.91/ F=27 & M=5	Placebo= 2.35 (0.13) ALN= 2.26 (0.23)	Placebo= 2.23 (0.13) ALN= 1.92 (0.23)	Parker et al. (16)
		Neridronate IV = 54	Neridronate IV=64±8/F	Neridronate IV = 2.6 (0.4)	Neridronate IV = 2.48 (0.40)	Rossini et al. (14)
		Placebo= 10 ALN= 12	Placebo=63.2±8.3/F ALN=69.4±6.3/F	Placebo= 2.70 (0.20) ALN=2.80 (0.45)	Placebo= 2.60 (0.35) ALN= 2.50 (0.18)	Akbaba et al. (19)
		Placebo= 15 ALN= 15	Placebo=57±5/F ALN=59±5/F	Placebo= 3.90 (0.30) ALN= 3.80 (0.20)	Placebo= 3.70 (0.40) ALN= 3.80 (0.30)	Cesareo et al. (24)
		Placebo= 15 Denosumab= 16	Placebo=68·0 ±1·8/ M=3, F=12 Denosumab= 65·4±2·2/ M=3, F=13	Placebo= 2.45 (0.09) Denosumab= 2.38 (0.74)	Placebo= 2.57 (0.15) Denosumab= 2.29 (0.87)	Leere et al. (21)
		Denosumab= 19	Denosumab=71.8 ± 7.1/ M=2, F=17	Denosumab= 3.20 (0.50)	Denosumab= 3 (0.60)	Miyaoka et al. (25)
		PTX= 24	PTX= 61.4±9.8/ M=7, F=17	PTX= 2.5 (0.7)	PTX= 3.2 (0.5)	Choe et al. (22)
	24 months	Placebo= 13 ALN= 13	Placebo=74±4/ F ALN=72±5/F	Placebo= 2.60 (0.50) ALN= 2.90 (0.60)	Placebo= 2.61 (0.62) ALN= 2.94 (0.66)	Rossini et al. (23)
		Placebo= 18 ALN= 14 After 24 months Placebo= 13 ALN= 10	Placebo= 63.4± 2.02/ ALN= 69.6± 2.91/ F=27 & M=5	Placebo= 2.35 (0.13) ALN= 2.26 (0.23)	Placebo= 2.20 (0.11) ALN= 2.35 (0.20)	Parker et al. (16)
		Neridronate IV = 54	Neridronate IV=64±8/F	Neridronate IV = 2.6 (0.4)	Neridronate IV = 2.57 (0.40)	Rossini et al. (14)
Osteocalcin (ng/mL)	12 months	Placebo= 13 ALN= 13	Placebo=74±4/ F ALN=72±5/F	Placebo= 3.60 (1.60) ALN= 4.10 (1.30)	Placebo= 3.67 (1.67) ALN= 2.81 (1.35)	Rossini et al. (23)
		Placebo= 18 ALN= 14	Placebo= 63.4± 2.02/ ALN= 69.6± 2.91/ F=27 & M=5	Placebo= 9.52 (1.40) ALN= 6.98 (1.40)	Placebo= 7.38 (1.53) ALN= 4.06 (1.44)	Parker et al. (16)
		Placebo= 20 ALN= 20	Placebo= 71.8± 8.8/F ALN= 68.2± 9.7/F	Placebo= 43.60 (28.5) ALN= 53 (28.90)	Placebo= 46.59 (7.86) ALN= 26.96 (4.32)	Chow et al. (17)
		ALN= 33 PTX= 33	F=60, M=3 54 post-menopausal, 6 pre-menopausal	ALN= 31 (13.90) PTX= 61.90 (75.70)	ALN= 26.05 (11.40) PTX= 16.50 (5.80)	Szymczak et al. (20)
	24 months	Placebo= 13 ALN= 13	Placebo=74±4/ F ALN=72±5/F	Placebo= 3.60 (1.60) ALN= 4.10 (1.30)	Placebo= 3.45 (1.68) ALN= 2.65 (1.36)	Rossini et al. (23)
		Placebo= 18 ALN= 14 After 24 months Placebo= 13 ALN= 10	Placebo= 63.4± 2.02/ ALN= 69.6± 2.91/ F=27 & M=5	Placebo= 9.52 (1.40) ALN= 6.98 (1.40)	Placebo= 8.01 (1.04) ALN= 6.44 (1.26)	Parker et al. (16)
BALP(U/L)	12 months	Placebo= 13 ALN= 13	Placebo=74±4/ F ALN=72±5/F	Placebo= 43 (9) ALN= 42 (12)	Placebo= 45 (9.45) ALN= 26.57 (12.5)	Rossini et al. (14)
		Placebo= 18 ALN= 14	Placebo= 63.4± 2.02/ ALN= 69.6± 2.91/ F=27 & M=5	Placebo= 136.53 (22.32) ALN= 147.59 (40.97)	Placebo= 113.38 (22.27) ALN= 58.08 (39.18)	Parker et al. (16)
		Placebo= 20 ALN= 20	Placebo= 71.8± 8.8/F ALN= 68.2± 9.7/F	Placebo= 21.80 (15.9) ALN= 21.10 (12.8)	Placebo= 23.38 (3.23) ALN= 7.26 (0.96)	Chow et al. (17)
		Neridronate IV = 54	Neridronate IV=64±8/F	Neridronate IV = 35 (14)	Neridronate IV = 25.86 (14.67)	Rossini et al. (14)
	24 months	Placebo= 13 ALN= 13	Placebo=74±4/ F ALN=72±5/F	Placebo= 43 (9) ALN= 42 (12)	Placebo= 43.69 (9.43) ALN= 25.42 (12.5)	Rossini et al. (23)
		Placebo= 18 ALN= 14 After 24 months Placebo= 13 ALN= 10	Placebo= 63.4± 2.02/ ALN= 69.6± 2.91/ F=27 & M=5	Placebo= 136.53 (22.32) ALN= 147.59 (40.97)	Placebo= 123.17 (11.57) ALN= 80.95 (30)	Parker et al. (16)
		Neridronate IV = 54	Neridronate IV=64±8/F	Neridronate IV = 35 (14)	Neridronate IV = 29.18 (14.74)	Rossini et al. (14)
CTX-I (ng/mL)	6 months	Neridronate IV = 54	Neridronate IV=64±8/F	Neridronate IV = 0.74 (0.39)	Neridronate IV = 0.47 (0.42)	Rossini et al. (14)
		Placebo= 15 ALN= 15	Placebo=57±5/F ALN=59±5/F	Placebo= 0.70 (0.10) ALN= 0.60 (0.10)	Placebo= 0.69 (0.10) ALN= 0.29 (0.05)	Cesareo et al. (24)
		ALN= 33 PTX= 33	F=60, M=3 54 post-menopausal, 6 pre-menopausal	ALN= 4.90 (2.03) PTX= 5.77 (4.5)	ALN= 4.55 (1.74) PTX= 3.58 (1.19)	Szymczak et al. (20)
	12 months	Neridronate IV = 54	Neridronate IV=64±8/F	Neridronate IV = 0.74 (0.39)	Neridronate IV = 0.45 (0.42)	Rossini et al. (14)
		ALN= 33 PTX= 33	F=60, M=3 54 post-menopausal, 6 pre-menopausal	ALN= 4.90 (2.03) PTX= 5.77 (4.5)	ALN= 4.21 (1.58) PTX= 3.70 (1.68)	Szymczak et al. (20)
		PTX= 24	PTX= 61.4±9.8/ M=7, F=17	PTX= 1.08 (0.91)	PTX= 0.20 (0.14)	Choe et al. (22)
	24 months	Neridronate IV = 54	Neridronate IV=64±8/F	Neridronate IV = 0.74 (0.39)	Neridronate IV = 0.38 (0.41)	Rossini et al. (14)
BMD (g/cm^2^) Whole body	12 months	Placebo= 13 ALN= 13	Placebo=74±4/ F ALN=72±5/F	Placebo= 0.82 (0.10) ALN= 0.82 (0.06)	Placebo= 0.81 (0.10) ALN= 0.83 (0.06)	Rossini et al. (23)
		EHDP= 9 PTX= 13	EHDP=76.3±5.2/F PTX=76.8±10.1/F	EHDP= 1.41 (0.23) PTX= 1.52 (0.26)	EHDP= 1.53 (0.20) PTX= 1.57 (0.32)	Horiuchi et al. (15)
		ALN= 33 PTX= 33	F=60, M=3 54 post-menopausal, 6 pre-menopausal	ALN= 1.00 (0.10) PTX= 0.92 (0.10)	ALN= 1.02 (0.12) PTX= 0.962 (0.1)	Szymczak et al. (20)
	24 months	Placebo= 13 ALN= 13	Placebo=74±4/ F ALN=72±5/F	Placebo= 0.82 (0.10) ALN= 0.82 (0.06)	Placebo= 0.80 (0.09) ALN= 0.83 (0.06)	Rossini et al. (23)
Lumbar spine	12 months	Placebo= 13 ALN= 13	Placebo=74±4/ F ALN=72±5/F	Placebo= 0.73 (0.07) ALN= 0.70 (0.09)	Placebo= 0.74 (0.07) ALN= 0.75 (0.09)	Rossini et al. (23)
		EHDP= 9 PTX= 13	EHDP=76.3±5.2/F PTX=76.8±10.1/F	EHDP= 0.62 (0.11) PTX= 0.69 (0.21)	EHDP= 0.68 (0.15) PTX= 0.83 (0.23)	Horiuchi et al. (15)
		Placebo= 18 ALN= 14	Placebo= 63.4± 2.02/ ALN= 69.6± 2.91/ F=27 & M=5	Placebo= 0.92 (0.08) ALN= 0.76 (0.07)	Placebo= 0.95 (0.04) ALN= 0.81 (0.11)	Parker et al. (16)
		Placebo= 20 ALN= 20	Placebo= 71.8± 8.8/F ALN= 68.2± 9.7/F	Placebo= 0.71 (0.15) ALN= 0.71 (0.12)	Placebo= 0.71 (0.15) ALN= 0.74 (0.12)	Chow et al. (17)
		Placebo= 19 ALN= 18	Placebo=70.09±10.36/M=6, F=13 ALN=63.73±9.36/ M=3, F=15	Placebo= 0.83 (0.13) ALN= 0.76 (0.11)	Placebo= 0.83 (0.14) ALN= 0.80 (0.12)	Khan et al. (18)
		Placebo= 10 ALN= 12	Placebo=63.2±8.3/F ALN=69.4±6.3/F	ALN= 0.57 (0.13)	ALN= 0.61 (0.12)	Akbaba et al. (19)
		ALN= 33 PTX= 33	F=60, M=3 54 post-menopausal, 6 pre-menopausal	ALN= 1.02 (0.17) PTX= 0.93 (0.20)	ALN= 1.04 (0.22) PTX= 0.99 (0.15)	Szymczak et al. (20)
		Denosumab= 7	Denosumab= 69.8 (range 62 – 81)/ Gender not given	Denosumab= 0.79 (0.11) (6 months)	Denosumab= 0.82 (0.13)	Grigorie et al. (26)
		Placebo= 15 ALN= 15	Placebo=57±5/F ALN=59±5/F	Placebo= 0.77 (0.07) ALN= 0.78 (0.07)	Placebo= 0.76 (0.07) ALN= 0.81 (0.07)	Cesareo et al. (24)
		Placebo= 15 Denosumab= 16	Placebo=68·0 ±1·8/ M=3, F=12 Denosumab= 65·4±2·2/ M=3, F=13	Denosumab= 0.83 (0.02)	Denosumab= 0.88 (0.12)	Leere et al. (21)
		Denosumab= 19	Denosumab=71.8 ± 7.1/ M=2, F=17	Denosumab= 0.73 (0.03)	Denosumab= 0.77 (0.03)	Miyaoka et al. (25)
		PTX= 24	PTX= 61.4±9.8/ M=7, F=17	PTX= 0.76 (0.08)	PTX= 0.89 (0.12)	Choe et al. (22)
	24 months	Placebo= 13 ALN= 13	Placebo=74±4/ F ALN=72±5/F	Placebo= 0.73 (0.07) ALN= 0.70 (0.09)	Placebo= 0.73 (0.07) ALN= 0.76 (0.09)	Rossini et al. (23)
		Placebo= 18 ALN= 14	Placebo= 63.4± 2.02/ ALN= 69.6± 2.91/ F=27 & M=5	Placebo= 0.92 (0.08) ALN= 0.76 (0.07)	Placebo= 0.96 (0.08) ALN= 0.85 (0.07)	Parker et al. (16)
		Placebo= 19 ALN= 18	Placebo=70.09±10.36/M=6, F=13 ALN=63.73±9.36/ M=3, F=15	Placebo= 0.83 (0.13) ALN= 0.76 (0.11)	ALN= 0.81 (0.12)	Khan et al. (18)
Femoral neck	12 months	Placebo= 13 ALN= 13	Placebo=74±4/ F ALN=72±5/F	Placebo= 0.55 (0.04) ALN= 0.58 (0.06)	Placebo= 0.55 (0.04) ALN= 0.59 (0.06)	Rossini et al. (23)
		Placebo= 18 ALN= 14	Placebo= 63.4± 2.02/ ALN= 69.6± 2.91/ F=27 & M=5	Placebo= 0.70 (0.04) ALN= 0.52 (0.07)	Placebo= 0.69 (0.08) ALN= 0.54 (0.04)	Parker et al. (16)
		Placebo= 20 ALN= 20	Placebo= 71.8± 8.8/F ALN= 68.2± 9.7/F	Placebo= 0.54 (0.12) ALN= 0.54 (0.11)	Placebo= 0.54 (0.12) ALN= 0.54 (0.11)	Chow et al. (17)
		Placebo= 19 ALN= 18	Placebo=70.09±10.36/M=6, F=13 ALN=63.73±9.36/ M=3, F=15	Placebo= 0.62 (0.12) ALN= 0.59 (0.10)	Placebo= 0.61 (0.12) ALN= 0.61 (0.10)	Khan et al. (18)
		Placebo= 10 ALN= 12	Placebo=63.2±8.3/F ALN=69.4±6.3/F	ALN= 0.74 (0.1)	ALN= 0.77 (0.08)	Akbaba et al. (19)
		ALN= 33 PTX= 33	F=60, M=3 54 post-menopausal, 6 pre-menopausal	ALN= 0.81 (0.12) PTX= 0.75 (0.10)	ALN= 0.83 (0.13) PTX= 0.79 (0.11)	Szymczak et al. (20)
		Denosumab= 7	Denosumab= 69.8 (range 62 – 81)/ Gender not given	Denosumab= 0.68 (0.07)	Denosumab= 0.69 (0.06)	Grigorie et al. (26)
		Placebo= 15 ALN= 15	Placebo=57±5/F ALN=59±5/F	Placebo= 0.64 (0.08) ALN= 0.62 (0.10)	Placebo= 0.63 (0.08) ALN= 0.64 (0.09)	Cesareo et al. (24)
		Placebo= 15 Denosumab= 16	Placebo=68·0 ±1·8/ M=3, F=12 Denosumab= 65·4±2·2/ M=3, F=13	Denosumab= 0.64 (0.08)	Denosumab= 0.67 (0.11)	Leere et al. (21)
		Denosumab= 19	Denosumab=71.8 ± 7.1/ M=2, F=17	Denosumab= 0.51 (0.02)	Denosumab= 0.53 (0.02)	Miyaoka et al. (25)
		PTX= 24	PTX= 61.4±9.8/ M=7, F=17	PTX= 0.68 (0.10)	PTX= 0.77 (0.12)	Choe et al. (22)
	24 months	Placebo= 13 ALN= 13	Placebo=74±4/ F ALN=72±5/F	Placebo= 0.55 (0.04) ALN= 0.58 (0.06)	Placebo= 0.55 (0.04) ALN= 0.59 (0.06)	Rossini et al. (23)
		Placebo= 18 ALN= 14	Placebo= 63.4± 2.02/ ALN= 69.6± 2.91/ F=27 & M=5	Placebo= 0.70 (0.04) ALN= 0.52 (0.07)	Placebo= 0.69 (0.04) ALN= 0.55 (0.07)	Parker et al. (16)
		Placebo= 19 ALN= 18	Placebo=70.09±10.36/M=6, F=13 ALN=63.73±9.36/ M=3, F=15	Placebo= 0.62 (0.12) ALN= 0.59 (0.10)	ALN= 0.62 (0.10)	Khan et al. (18)
Total hip	12 months	Placebo= 13 ALN= 13	Placebo=74±4/ F ALN=72±5/F	Placebo= 0.57 (0.08) ALN= 0.61 (0.07)	Placebo= 0.56 (0.08) ALN= 0.63 (0.07)	Rossini et al. (23)
		Placebo= 19 ALN= 18	Placebo=70.09±10.36/M=6, F=13 ALN=63.73±9.36/ M=3, F=15	Placebo= 0.72 (0.14) ALN= 0.67 (0.14)	Placebo= 0.72 (0.14) ALN= 0.70 (0.14)	Khan et al. (18)
		Placebo= 15 Denosumab= 16	Placebo=68·0 ±1·8/ M=3, F=12 Denosumab= 65·4±2·2/ M=3, F=13	Denosumab= 0.78 (0.08)	Denosumab= 0.82 (0.11)	Leere et al. (21)
		Denosumab= 19	Denosumab=71.8 ± 7.1/ M=2, F=17	Denosumab= 0.63 (0.02)	Denosumab= 0.64 (0.03)	Miyaoka et al. (25)
		PTX= 24	PTX= 61.4±9.8/ M=7, F=17	PTX= 0.71 (0.12)	PTX= 0.80 (0.13)	Choe et al. (22)
		Placebo= 20 ALN= 20	Placebo= 71.8± 8.8/F ALN= 68.2± 9.7/F	Placebo=0.49 (0.09) ALN=0.47 (0.08)	Placebo= 0.49 (0.09) ALN= 0.47 (0.08)	Chow et al. (17)
Distal radius (1/3)	12 months	Placebo= 19 ALN= 18	Placebo=70.09±10.36/M=6, F=13 ALN=63.73±9.36/ M=3, F=15	Placebo= 0.55 (0.12) ALN= 0.52 (0.11)	Placebo= 0.55 (0.12) ALN= 0.52 (0.12)	Khan et al. (18)
		Placebo= 10 ALN= 12	Placebo=63.2±8.3/F ALN=69.4±6.3/F	ALN= 0.72 (0.18)	ALN= 0.69 (0.61)	Akbaba et al. (19)
		Placebo= 15 Denosumab= 16	Placebo=68·0 ±1·8/ M=3, F=12 Denosumab= 65·4±2·2/ M=3, F=13	Denosumab= 0.57 (0.08)	Denosumab= 0.58 (0.10)	Leere et al. (21)

# F, female; M, male; ALN, alendronate; EHDP, etidronate; PTX, parathyroidectomy.

### 2.4 Outcome assessment

Based on the type of drugs used, studies were categorized into the following groups: BP, denosumab, and PTX. In the present study, bone loss was assessed by aBMD and BTMs (OCN, BALP, and CTX-I). The effect of various drugs in PHPT patients were assessed on serum PTH, calcium, and phosphate levels.

### 2.5 Quantitative data analysis

Cochrane’s Q test determined the degree of heterogeneity among the studies and heterogeneity index (I^2^), considering p value < 0.05 as statistically significant. The I^2^ value lies between 0 and 100%; I^2^> 75% indicates high heterogeneity, I^2^> 50% indicates moderate heterogeneity, and I^2^< 25% suggests low heterogeneity. Significant heterogeneity favors the use of the random effects model, while low heterogeneity favors the use of the fixed effect model. The comprehensive meta-analysis software (CMA) was used to perform the pooled data analysis.

### 2.6 Sensitivity analysis

The CMA software was used to determine the degree of sensitivity among these studies. The pooled effect size was determined using single-study exclusion statistics to identify the sensitive studies, the exclusion of which would bring drastic changes to the inference.

### 2.7 Publication bias analysis

Publication bias was assessed qualitatively and quantitatively using funnel plot and Egger’s regression intercept test and Begg and Mazumdar rank correlation test, respectively. Publication bias was adjusted by calculating unbiased estimates using Duval and Tweedie’s trim and fill method.

## 3 Results

### 3.1 Study characteristics and quality

A literature search for the effect of BPs and denosumab on PHPT-induced bone loss identified 3,891 articles: Scopus (3,320), PubMed (559), and Cochrane library (12). After the removal of duplicates, 1914 articles were screened. Out of these, 1,895 studies were excluded based on the set inclusion/exclusion criteria, and 19 were selected for qualitative analysis. Of the selected studies, 14 studies reported the use of BPs in PHPT patients. Of these, 8 studies were done with alendronate (ALN) (16–20, 23, 24, 27), and one each with etidronate (15), neridronate (14), and risedronate (28). Four studies (27–30) were excluded from this meta-analysis because of the following reasons; the types of BP were not mentioned, data were not presented in the required format, and the treatment duration was five years. Taken together, for ALN trials, 7 studies used only ALN, and one used a combination of BP and PTX for PHPT patients (22).

There were five studies in which denosumab was given to PHPT patients, of which two were excluded because data were not presented in the required format (31, 32). The remaining three studies were included in the meta-analysis (21, 25, 26). There were two studies in which the effect of BPs was compared with PTX (15, 20) and one study in which combination of BP and PTX was compared with PTX (22) in PHPT patients. These three studies were included in determining the effect of PTX on BMD and serum PTH in PHPT patients. In total, 13 studies were finally included for quantitative meta-analysis (**Figure 1**). A summary of all the results for various parameters is shown in **Table 2**.

**Table 2:** The summary of pooled analysis for various parameters.

Groups	Parameters	Heterogeneity analysis			Test model	Effect size			p-value	Conclusion
						SDM	95% CI			
		Q	p-value	I^2^			Lower limit	Upper limit		
										
**12 months ALN use compared with Placebo**	Lumbar spine BMD	1.892	0.756	0.000	**Fixed**	**0.350**	**0.041**	**0.659**	**0.027**	Significant
					Random	0.350	0.041	0.659	0.027	
	Femur neck BMD	2.538	0.638	0.000	**Fixed**	**0.250**	**-0.058**	**0.558**	**0.111**	Non-significant
					Random	0.250	-0.058	0.558	0.111	
	PTH	11.574	0.041	56.799	Fixed	0.612	0.313	0.911	0.0001	Significant
					**Random**	**0.602**	**0.145**	**1.059**	**0.010**	
	Calcium (Ca)	32.902	0.000	87.843	Fixed	-0.414	-0.749	-0.079	0.015	Non-significant
					**Random**	**-0.381**	**-1.345**	**0.583**	**0.439**	
	Phosphate	12.232	0.007	75.474	Fixed	-0.338	-0.727	0.050	0.088	Non-significant
					**Random**	**-0.369**	**-1.156**	**0.418**	**0.358**	
	OCN	35.212	0.000	94.320	Fixed	-1.346	-1.831	-0.860	0.0001	Non-significant
					**Random**	**-1.947**	**-4.064**	**0.170**	**0.072**	
	BALP	31.008	0.000	93.550	Fixed	-2.552	-3.134	-1.970	0.0001	Significant
					**Random**	**-3.422**	**-5.844**	**-1.000**	**0.006**	
									
**12 months anti-resorptives use compared with baseline**	Lumbar spine BMD	8.880	0.448	0.000	**Fixed**	**0.447**	**0.230**	**0.664**	**0.0001**	Significant
					Random	0.447	0.230	0.664	0.0001	
	Femur neck BMD	5.748	0.675	0.000	**Fixed**	**0.270**	**0.049**	**0.491**	**0.017**	Significant
					Random	0.270	0.049	0.491	0.017	
	Total hip BMD	0.261	0.967	0.000	**Fixed**	**0.330**	**-0.014**	**0.673**	**0.060**	Non-Significant
					Random	0.330	-0.014	0.673	0.060	
	Distal radius BMD	0.097	0.992	0.000	**Fixed**	**0.042**	**-0.300**	**0.383**	**0.810**	Non-significant
					Random	0.042	-0.300	0.383	0.810	
	PTH	24.599	0.003	63.413	Fixed	0.363	0.165	0.562	0.0001	Significant
					**Random**	**0.489**	**0.139**	**0.839**	**0.006**	
	Calcium	18.785	0.009	62.736	Fixed	-0.471	-0.696	-0.245	0.0001	Significant
					**Random**	**-0.545**	**-0.937**	**-0.154**	**0.006**	
	Phosphate	10.834	0.094	44.617	**Fixed**	**-0.357**	**-0.594**	**-0.120**	**0.003**	Significant
					Random	-0.393	-0.733	-0.054	0.023	
									
**12 months BP use compared with baseline**	Total BMD	0.506	0.776	0.000	**Fixed**	**0.235**	**-0.141**	**0.610**	**0.221**	Non-significant
					Random	0.235	-0.141	0.610	0.221	
	Lumbar spine BMD	1.774	0.971	0.000	**Fixed**	**0.330**	**0.088**	**0.571**	**0.007**	Significant
					Random	0.330	0.088	0.571	0.007	
	Femur neck	0.622	0.996	0.000	**Fixed**	**0.170**	**-0.079**	**0.418**	**0.181**	Non-significant
					Random	0.170	-0.079	0.418	0.181	
	PTH	23.808	0.002	66.398	Fixed	0.390	0.183	0.598	0.0001	Significant
					**Random**	**0.546**	**0.162**	**0.930**	**0.005**	
	Calcium	17.753	0.007	66.203	Fixed	-0.511	-0.749	-0.273	0.0001	Significant
					**Random**	**-0.608**	**-1.048**	**-0.169**	**0.007**	
	Phosphate	10.296	0.036	61.148	Fixed	-0.394	-0.668	-0.120	0.005	Non-significant
					**Random**	**-0.478**	**-0.969**	**0.012**	**0.056**	
	Osteocalcin	10.967	0.012	72.645	Fixed	-0.901	-1.234	-0.568	0.0001	Significant
					**Random**	**-1.097**	**-1.774**	**-0.420**	**0.001**	
	BALP	12.372	0.006	75.751	Fixed	-1.035	-1.333	-0.736	0.0001	Significant
					**Random**	**-1.339**	**-2.035**	**-0.643**	**0.0001**	
	CTX-I (6months)	30.992	0.000	93.547	Fixed	-0.677	-0.971	-0.383	0.0001	Significant
					**Random**	**-1.417**	**-2.741**	**-0.092**	**0.036**	
									
**Denosumab use compared with baseline**	Lumbar spine BMD	3.526	0.172	43.283	**Fixed**	**0.828**	**0.378**	**1.278**	**0.0001**	Significant
					Random	0.793	0.179	1.407	0.011	
	Femur neck BMD	2.664	0.264	24.937	**Fixed**	**0.575**	**0.135**	**1.015**	**0.010**	Significant
					Random	0.559	0.042	1.077	0.034	
									
**Parathyroidectomy compared with baseline**	Lumbar spine BMD	4.550	0.103	56.045	**Fixed**	**0.662**	**0.319**	**1.005**	**0.0001**	Significant
					Random	0.700	0.162	1.238	0.011	
	PTH	28.090	0.000	92.880	Fixed	-1.513	-1.909	-1.117	0.0001	Significant
					**Random**	**-2.723**	**-4.466**	**-0.980**	**0.002**	
									
**12 months ALN use compared with baseline**	Lumbar spine BMD	1.701	0.945	0.000	**Fixed**	**0.321**	**0.071**	**0.571**	**0.012**	Significant
					Random	0.321	0.071	0.571	0.012	
	Femur neck BMD	0.622	0.996	0.000	**Fixed**	**0.170**	**-0.079**	**0.418**	**0.181**	Non-significant
					Random	0.170	-0.079	0.418	0.181	
	Distal radius	0.048	0.976	0.000	**Fixed**	**0.020**	**-0.372**	**0.412**	**0.921**	Non-significant
					Random	0.020	-0.372	0.412	0.921	
	PTH	5.620	0.46 7	0.000	**Fixed**	**0.416**	**0.164**	**0.668**	**0.001**	Significant
					Random	0.416	0.164	0.668	0.001	
	Calcium	14.201	0.007	71.833	Fixed	-0.618	-0.949	-0.287	0.0001	Significant
					**Random**	**-0.632**	**-1.261**	**-0.004**	**0.049**	
	Phosphate	9.732	0.021	69.175	Fixed	-0.504	-0.899	-0.108	0.013	Non-significant
					**Random**	**-0.567**	**-1.283**	**0.150**	**0.121**	
	Osteocalcin	10.967	0.012	72.645	Fixed	-0.901	-1.234	-0.568	0.0001	Significant
					**Random**	**-1.097**	**-1.774**	**-0.420**	**0.001**	
	BALP	2.394	0.302	16.475	**Fixed**	**-1.617**	**-2.086**	**-1.149**	**0.0001**	Significant
					Random	-1.625	-2.142	-1.109	0.0001	
									
**24 months ALN use compared with baseline**	Lumbar spine BMD	2.057	0.358	2.766	**Fixed**	**0.724**	**0.295**	**1.153**	**0.001**	Significant
					Random	0.726	0.290	1.161	0.001	
	Femur neck BMD	0.164	0.922	0.000	**Fixed**	**0.274**	**-0.141**	**0.690**	**0.195**	Non-significant
					Random	0.274	-0.141	0.690	0.195	
	PTH	6.091	0.048	67.163	Fixed	0.136	-0.296	0.569	0.537	Non-Significant
					**Random**	**0.213**	**-0.551**	**0.977**	**0.585**	
	Calcium	21.064	0.000	90.505	Fixed	-0.311	-0.767	0.145	0.181	Non-significant
					**Random**	**-0.245**	**-1.728**	**1.237**	**0.746**	

Bold values were used for drawing conclusion.

The majority of the studies used baseline control, although some of the ALN studies used placebo control. Therefore, baseline and placebo control were used separately in this meta-analysis.

### 3.2 Effects of anti-resorptive drugs on aBMD and biochemical parameters compared with baseline

**Figure 2:**
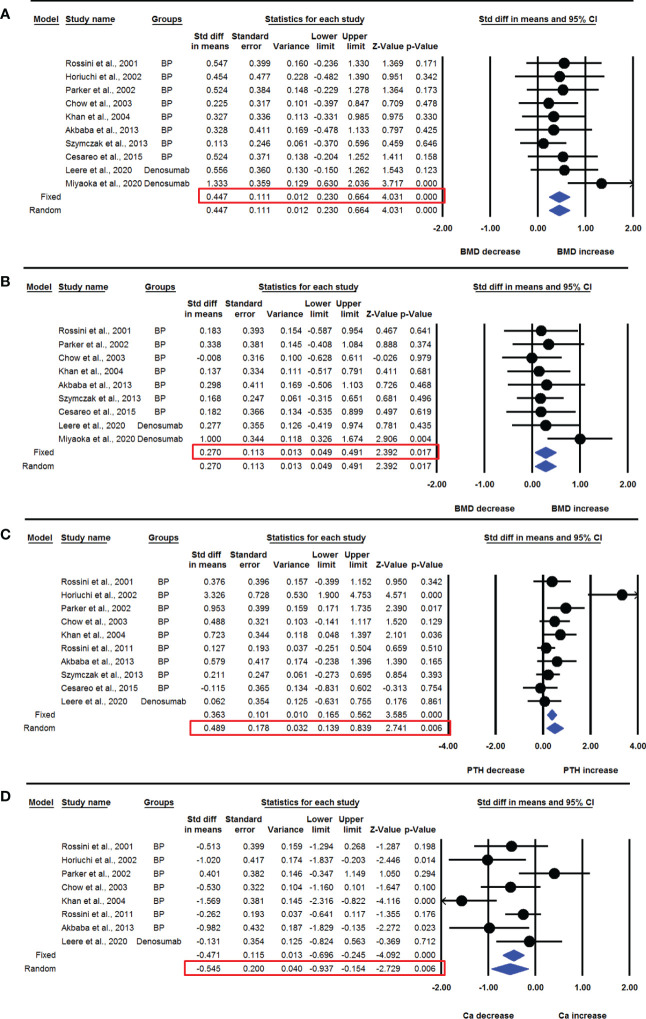
The effect of anti-resorptive drug administration on various parameters in PHPT patients compared with baseline; **(A)** lumbar spine aBMD, **(B)** femoral neck aBMD, **(C)** serum PTH, and **(D)** serum calcium (Ca).

We pooled data from the studies that used BPs or denosumab to determine the overall effect of anti-resorptive therapies on aBMD and serum parameters. Ten datasets from as many studies were available for lumbar spine aBMD before and 12 months after BP or denosumab use. The fixed effect model was used for drawing inference because there was no significant heterogeneity between the studies (I^2^=0.000, Q=8.880, p=0.448). Pooled analysis showed a significant increase in the mean lumbar spine aBMD after drug administration compared with baseline (SDM=0.447, 95% CI=0.230 to 0.664, p=0.0001) (**Figure 2A**).

Nine datasets from as many studies were available for femur neck aBMD before and 12 months after drug administration. No significant heterogeneity was observed among these studies (I^2^=0.000, Q=5.748, p=0.675), suggesting the use of fixed effect model. Pooled analysis showed that the mean femoral neck aBMD was significantly increased after 12 months of drug administration compared with baseline (SDM=0.270, 95% CI=0.049 to 0.491, p=0.017) (**Figure 2B**).

Four datasets from as many studies were available for total hip aBMD before and 12 months after BP or denosumab use. No significant heterogeneity was found among these studies (I^2^=0.000, Q=0.261, p=0.967), suggesting the use of fixed effect model for drawing inference. Pooled analysis showed no significant change in mean total hip aBMD after drug administration compared with baseline (SDM=0.330, 95% CI=-0.014 to 0.673, p=0.060) (**Supplementary Figure 1A**).

Four datasets from as many studies were available for aBMD of distal radius before and after 12 months of BP or denosumab use. No significant heterogeneity was observed among these studies (I^2^=0.000, Q=0.097, p=0.992), suggesting the use of fixed effect model for drawing a conclusion. There was no change in the mean distal radius aBMD after 12 months of drug use compared with baseline (SDM=0.042, 95% CI=-0.300 to 0.383, p=0.810) (**Supplementary Figure 1B**).

Ten datasets from as many studies were available for serum PTH levels before and 12 months after drug administration, including BP or denosumab. Significant heterogeneity was observed among these studies (I^2^=63.413, Q=24.599, p=0.003), which suggested using the random effects model to draw a conclusion. Pooled analysis showed a significant increase in mean PTH levels after drug administration compared with baseline (SDM=0.489, 95% CI=0.139 to 0.839, p=0.006) (**Figure 2C**). The funnel plot showed an asymmetric distribution of studies, suggesting the presence of publication bias (Egger’s regression intercept=3.618; p=0.018). So, we used trim and fill analysis to compute unbiased estimates and adjusted the values (SDM= 0.228, 95% CI=-0.172 to 0.628).

Eight datasets from as many studies were available for serum calcium levels before and 12 months after drug administration. The random effects model was used in the pooled analysis to draw a conclusion because significant heterogeneity was found in these studies (I^2^=62.736, Q=18.785, p=0.009). Pooled analysis showed a significant decrease in mean serum calcium levels after drug administration compared with baseline (SDM=-0.545, 95% CI=-0.937 to -0.154, p=0.006) (**Figure 2D**).

Seven datasets from as many studies were available for serum phosphate levels before and 12 months after drug use. No significant heterogeneity was observed among these studies (I^2^=44.617, Q=10.834, p=0.094), suggesting the use of fixed effect model for drawing inference. The pooled analysis showed that mean serum phosphate levels were significantly decreased after drug administration (SDM=-0.357, 95% CI=-0.594 to -0.120, p=0.003) (**Supplementary Figure 1C**).

### 3.3 Effects of BPs on aBMD and biochemical parameters compared with baseline

In the previous section, we found that anti-resorptive therapies improved aBMD at many sites while decreasing serum calcium level in PHPT patients. Here, we analyzed the effect of only BPs on aBMD and biochemical parameters. Three datasets from as many studies were available for total aBMD before and 12 months after BP use. Heterogeneity was not significant between these studies (I^2^=0.000, Q=0.506, p=0.776), suggesting the use of fixed effect model for drawing inference. There was no significant change in the mean total aBMD after BP administration compared with baseline (SDM=0.235, 95% CI=-0.141 to 0.610, p=0.221) (**Supplementary Figure 2A**).

**Figure 3:**
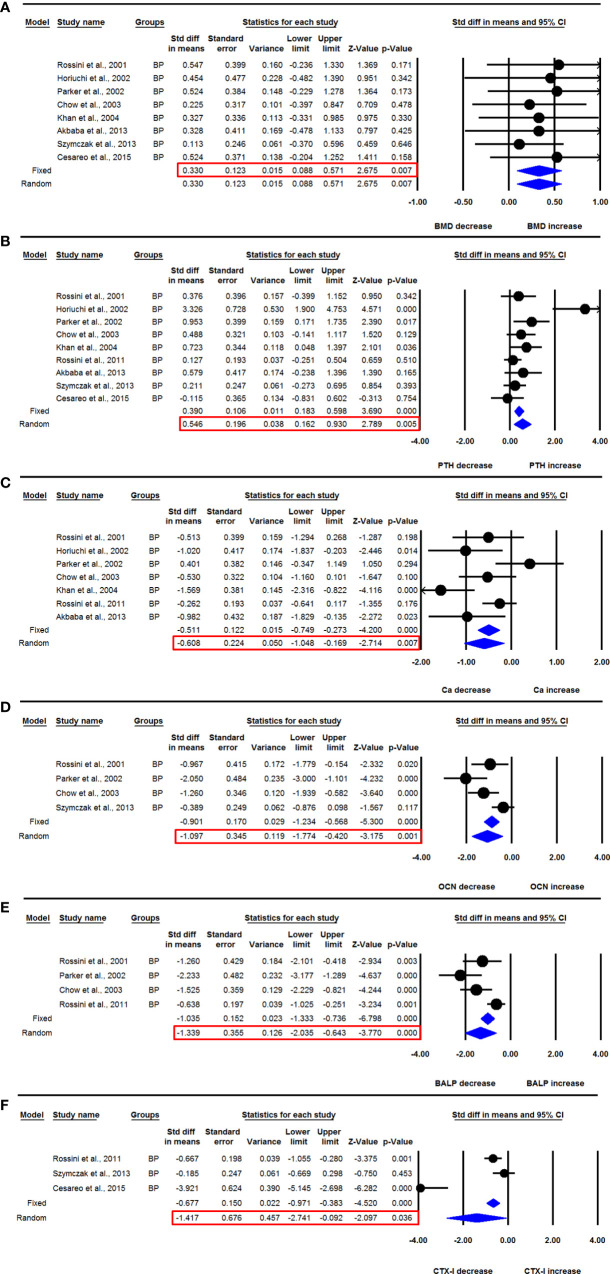
The effect of BP administration on various parameters in PHPT patients compared with baseline; **(A)** lumbar spine aBMD, **(B)** serum PTH, **(C)** serum calcium (Ca), **(D)** serum OCN, **(E)** serum BALP, and **(F)** serum CTX-I.

Eight datasets from as many studies were available for the lumbar spine aBMD before and 12 months after BP use. No significant heterogeneity was observed between these studies (I^2^=0.000, Q=1.774, p=0.971), suggesting the use of fixed effect model for drawing a conclusion. The mean lumbar spine aBMD was significantly increased after BP administration compared with baseline (SDM=0.330, 95% CI=0.088 to 0.571, p=0.007) (**Figure 3A**). Studies were distributed asymmetrically in the funnel plot (Egger’s regression intercept= 2.018, p= 0.009), suggesting the presence of publication bias. So, we used the adjusted values according to the trim and fill method for unbiased estimates (SDM=0.240, 95% CI=0.029 to 0.451).

Seven datasets from as many studies were available for femur neck aBMD before and 12 months after BP use. No significant heterogeneity was observed between these studies (I^2^=0.000, Q=0.622, p=0.996), suggesting the use of fixed effect model for data analysis. Pooled analysis indicated no significant change in mean femur neck aBMD after BP use (SDM=0.170, 95% CI=-0.079 to 0.418, p=0.181) (**Supplementary Figure 2B**).

Nine datasets from as many studies were available for serum PTH levels before and 12-months after BP use. Significant heterogeneity was found between these studies (I^2^=66.398, Q=23.808, p=0.002), suggesting that the random effects model should be used for drawing inference. Mean serum PTH levels were significantly increased after BP therapy in PHPT patients (SDM=0.546, 95% CI=0.162 to 0.930, p=0.005) (**Figure 3B**). The funnel plot showed an asymmetric distribution of studies (Egger’s regression intercept=3.832, p=0.016), suggesting the presence of publication bias. We used trim and fill analysis to compute unbiased estimates and adjusted the values (SDM= 0.250, 95% CI= -0.181 to 0.681).

Seven datasets from as many studies were available for serum calcium levels before and 12-months after BP use. The random effects model was used for drawing conclusion because significant heterogeneity was observed in these studies (I^2^=66.203, Q=17.753, p=0.007). Pooled analysis showed that the mean serum calcium level was significantly decreased after BP administration compared with baseline (SDM=-0.608, 95% CI=-1.048 to -0.169, p=0.007) (**Figure 3C**).

Five datasets from as many studies were available for serum phosphate levels before and 12-months after BP use. Significant heterogeneity was found in these studies (I^2^=61.148, Q=10.296, p=0.036), resulting in the use of the random effects model for data analysis. The pooled analysis showed no significant change in serum phosphate levels after BP administration (SDM=-0.478, 95% CI=-0.969 to -0.012, p=0.056) (**Supplementary Figure 2C**).

Four datasets from as many studies were available for serum OCN levels before and 12 months after BP use. I^2^ values showed that heterogeneity was significant in these studies (I^2^=72.645, Q=10.967, p=0.012), which suggested that the random effects model should be used for drawing a conclusion. Pooled analysis showed that the mean serum OCN levels were significantly decreased after BP use (SDM= -1.097, 95% CI=-1.774 to -0.420, p=0.001) (**Figure 3D**).

Four datasets from as many studies were available for serum BALP levels before and 12 months after BP use. There was significant heterogeneity between the studies (I^2^=75.751, Q=12.372, p=0.006), suggesting the use of random effects model for drawing inference. The mean serum BALP level was significantly reduced after BP administration compared with baseline (SDM=-1.339, 95% CI= -2.035 to -0.643, p=0.0001) (**Figure 3E**).

Three datasets from as many studies were available for serum CTX-1 before and 6 months after BP use. Significant heterogeneity was observed in these studies (I^2^=93.547, Q=30.992, p=0.000), suggesting the use of random effects model for data analysis. The mean serum CTX-1 level was significantly decreased after BP administration compared with baseline (SDM=-1.417, 95% CI=-2.741 to -0.092, p=0.036) (**Figure 3F**).

### 3.4 Effects of ALN on aBMD and biochemical parameters

#### 3.4.1 Comparison with the baseline values

In the previous section, we found that BP administration significantly improved aBMD, and decreased serum calcium as well as BTMs. Here, we analyzed the effect of ALN on PHPT patients because a sufficient number of studies was available to perform a meta-analysis.

**Figure 4:**
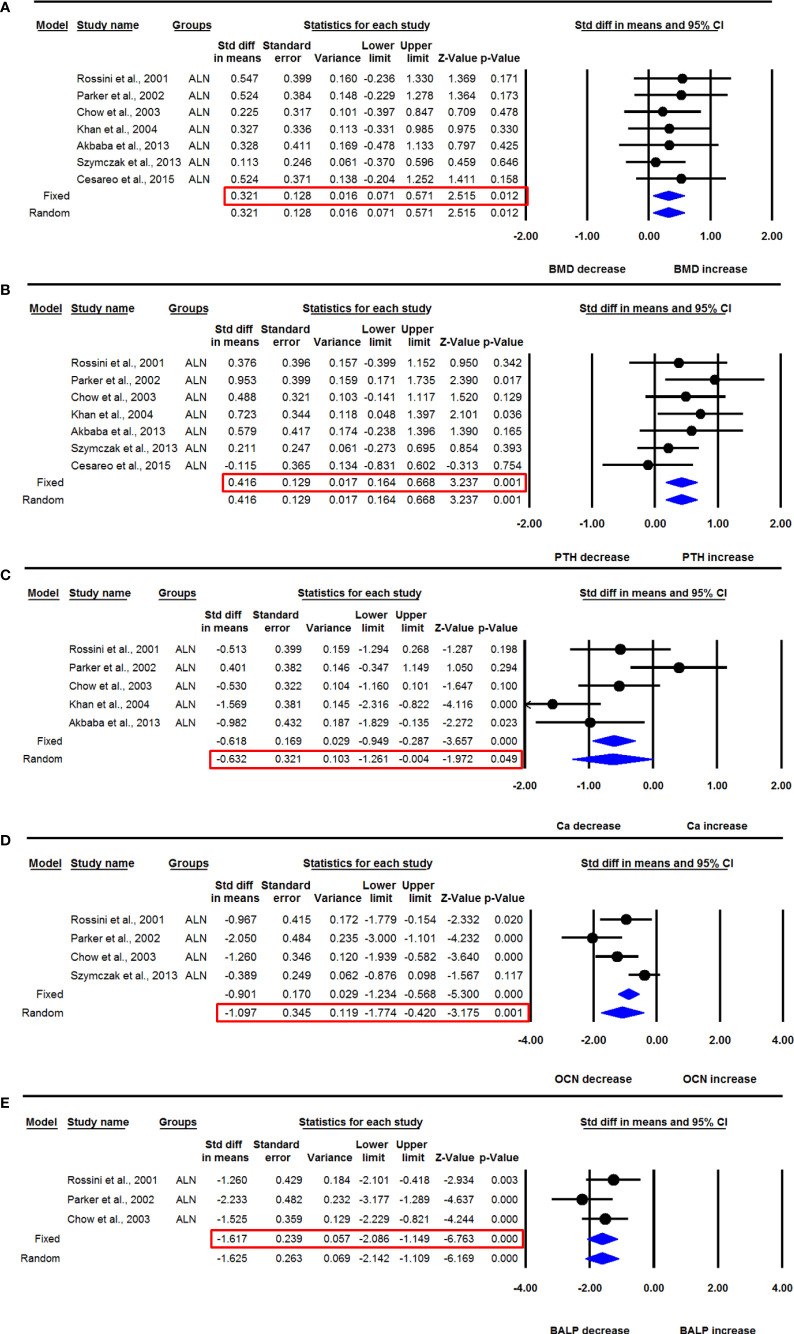
The effect of ALN administration in PHPT patients compared with baseline; **(A)** lumbar spine aBMD, **(B)** serum PTH, **(C)** serum calcium (Ca), **(D)** serum OCN, and **(E)** serum BALP.

Seven datasets from as many studies were available for lumbar spine aBMD before and 12 months after ALN use. The heterogeneity between these studies was not significant (I^2^=0.000, Q=1.701, p=0.945), suggesting the use of the fixed effect model for drawing inferences. Pooled analysis showed a significant increase in the mean lumbar spine aBMD after ALN administration compared with baseline (SDM=0.321, 95% CI=0.071 to 0.571, p=0.012) (**Figure 4A**). The funnel plot showed an asymmetric distribution of studies (Egger’s regression intercept=2.455, p=0.009), suggesting the presence of publication bias. Unbiased estimates were used from the trim and fill method, and the values were adjusted (SDM= 0.199, 95% CI=-0.011 to 0.408).

Seven datasets from as many studies were available for femoral neck aBMD before and 12 months after ALN use. I^2^ value showed no significant heterogeneity between these studies (I^2^=0.000, Q=0.622, p=0.996), suggesting the use of the fixed effect model for drawing conclusions. Pooled analysis showed that there was no significant change in the mean femoral neck aBMD after ALN administration (SDM=0.170, 95% CI=-0.079 to 0.418, p=0.181) (**Supplementary Figure 3A**).

Three datasets from as many studies were available for the distal radius aBMD before and 12 months after ALN administration. There was no significant heterogeneity between the studies (I^2^=0.000, Q=0.048, p=0.976), suggesting the use of the fixed effect model for data analysis. Pooled analysis showed no significant change in distal radius aBMD after ALN use in PHPT patients (SDM=0.020, 95% CI=-0.372 to 0.412, p=0.921) (**Supplementary Figure 3B**).

Seven datasets from as many studies were available for serum PTH before and 12 months after ALN administration. Since the heterogeneity between these studies was not significant (I^2^=0.000, Q=5.620, p=0.467), we used the fixed effect model for drawing inference. Pooled analysis showed a significant increase in serum PTH after ALN use (SDM=0.416, 95% CI=0.164 to 0.668, p=0.001) (**Figure 4B**).

Five datasets from as many studies were available for serum calcium before and 12 months after ALN use. There was significant heterogeneity between these studies (I^2^=71.833, Q=14.201, p=0.007), suggesting the use of the random effects model for drawing a conclusion. Pooled analysis showed a significant decrease in serum calcium after ALN use (SDM=-0.632, 95% CI=-1.261 to -0.004, p=0.049) (**Figure 4C**).

Four datasets from as many studies were available for serum phosphate before and 12 months after ALN use. Significant heterogeneity was found between these studies (I^2^=69.175, Q= 9.732, p=0.021), which suggested the use of the random effects model. Pooled analysis showed no significant change in serum phosphate after ALN use (SDM=-0.567, 95% CI=-1.283 to 0.150, p=0.121) (**Supplementary Figure 3C**).

Four datasets from as many studies were available for serum OCN before and 12 months after ALN use. Significant heterogeneity was found between these studies (I^2^=72.645, Q=10.967, p=0.012), suggesting the use of the random effects model for drawing a conclusion. Pooled analysis showed a significant decrease in serum OCN after ALN use (SDM=-1.097, 95% CI=-1.774 to -0.420, p=0.001) (**Figure 4D**).

Three datasets from as many studies were available for serum BALP before and 12 months after ALN use. The heterogeneity between these studies was not significant (I^2^=16.475, Q=2.394, p=0.302), suggesting the use of the fixed effect model for drawing inference. Pooled analysis showed a significant decrease in serum BALP after ALN use (SDM=-1.617, 95% CI=-2.086 to -1.149, p= 0.0001) (**Figure 4E**).

#### 3.4.2 Comparison with the placebo control

**Figure 5:**
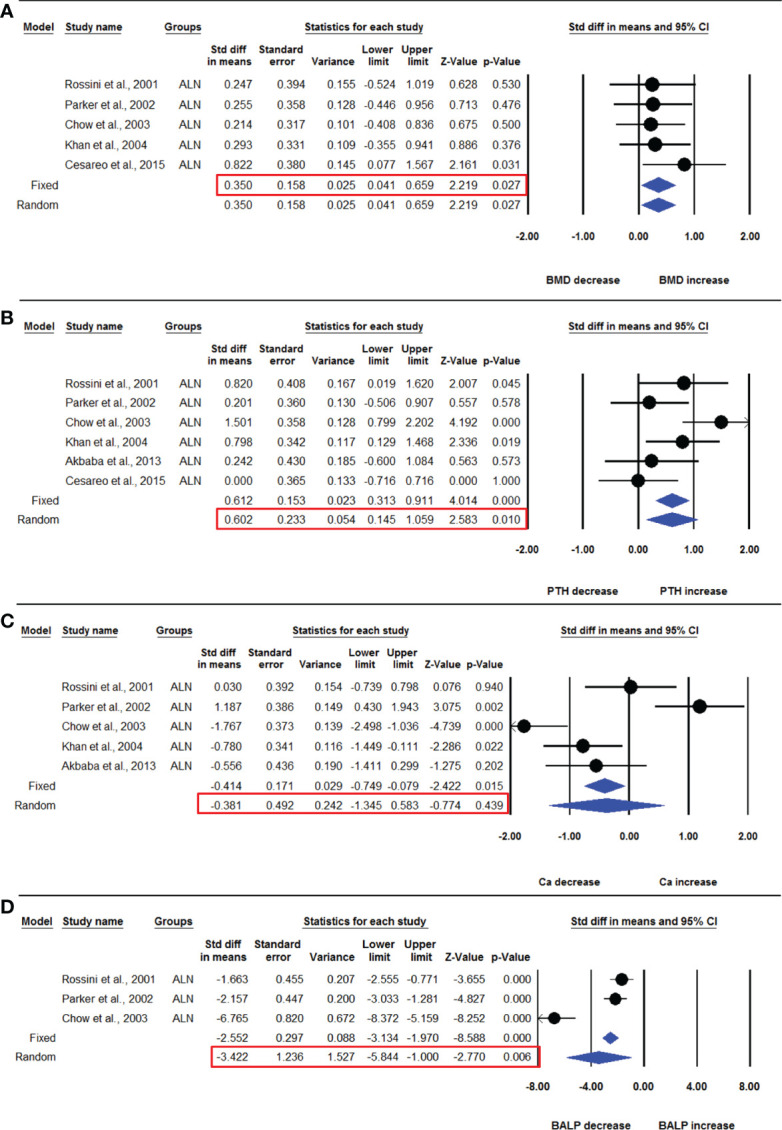
The effect of ALN administration on different parameters in PHPT patients compared with the placebo group; **(A)** lumbar spine aBMD, **(B)** serum PTH, **(C)** serum calcium (Ca), and **(D)** serum BALP.

Five datasets from as many studies were available for lumbar spine aBMD in PHPT patients treated with ALN for 12 months and compared with placebo control. No significant heterogeneity was found among these studies (I^2^=0.000, Q=1.892, p=0.756), suggesting that the fixed effect model should be used for data analysis. The pooled analysis showed that the mean lumbar spine aBMD in the ALN group was significantly increased compared with the placebo group (SDM=0.350, 95% CI=0.041 to 0.659, p=0.027) (**Figure 5A**).

Five datasets from as many studies were available for femoral neck aBMD in the ALN and placebo groups. No significant heterogeneity was observed among these studies (I^2^=0.000, Q=2.538, p=0.638), suggesting the use of the fixed effect model for drawing inference. The mean femoral neck aBMD was not significantly different in the ALN and placebo group (SDM=0.250, 95% CI=-0.058 to 0.558, p=0.111) (**Supplementary Figure 4A**).

Six datasets from as many studies were analyzed for serum PTH in the ALN and placebo groups. Significant heterogeneity was found in these studies (I^2^=56.799, Q=11.574, p=0.041), which suggested the use of the random effects model for drawing conclusions. The pooled analysis shows that PTH was significantly higher in the ALN group compared with placebo (SDM=0.602, 95% CI=0.145 to 1.059, p=0.010) (**Figure 5B**).

Five datasets from as many studies were available for serum calcium in the ALN and placebo group. I^2^ values showed significant heterogeneity (I^2^=87.843, Q=32.902, p=0.000), suggesting the use of the random effects model. The mean serum calcium was not different in the ALN and placebo groups (SDM=-0.381, 95% CI= -1.345 to 0.583, p=0.439) (**Figure 5C**).

Four datasets from as many studies were available for serum phosphate in both groups. Since no significant heterogeneity was found among these studies (I^2^=75.474, Q=12.232, p=0.007), we applied the random effects model to draw the inference. No significant change was found between the ALN and placebo groups (SDM=-0.369, 95% CI=-1.156 to 0.418, p=0.358) (**Supplementary Figure 4B**).

Three datasets from as many studies were available for serum BALP in the ALN and placebo groups. Significant heterogeneity was observed among these studies (I^2^=93.550, Q=31.008, p=0.000), so the random effects model was used for drawing conclusions. The mean BALP was significantly decreased in the ALN group compared with the placebo (SDM=-3.422, 95% CI=-5.844 to -1.000, p=0.006) (**Figure 5D**).

Three datasets from as many studies were available for serum OCN. Significant heterogeneity was found in these studies (I^2^=94.320, Q=35.212, p=0.000), suggesting the use of the random effects model for inference. The mean serum OCN was not significantly different in the ALN and placebo groups (SDM=-1.947, 95% CI=-4.064 to 0.170, p=0.072) (**Supplementary Figure 4C**).

### 3.5 Effect of denosumab on aBMD compared with baseline

**Figure 6:**
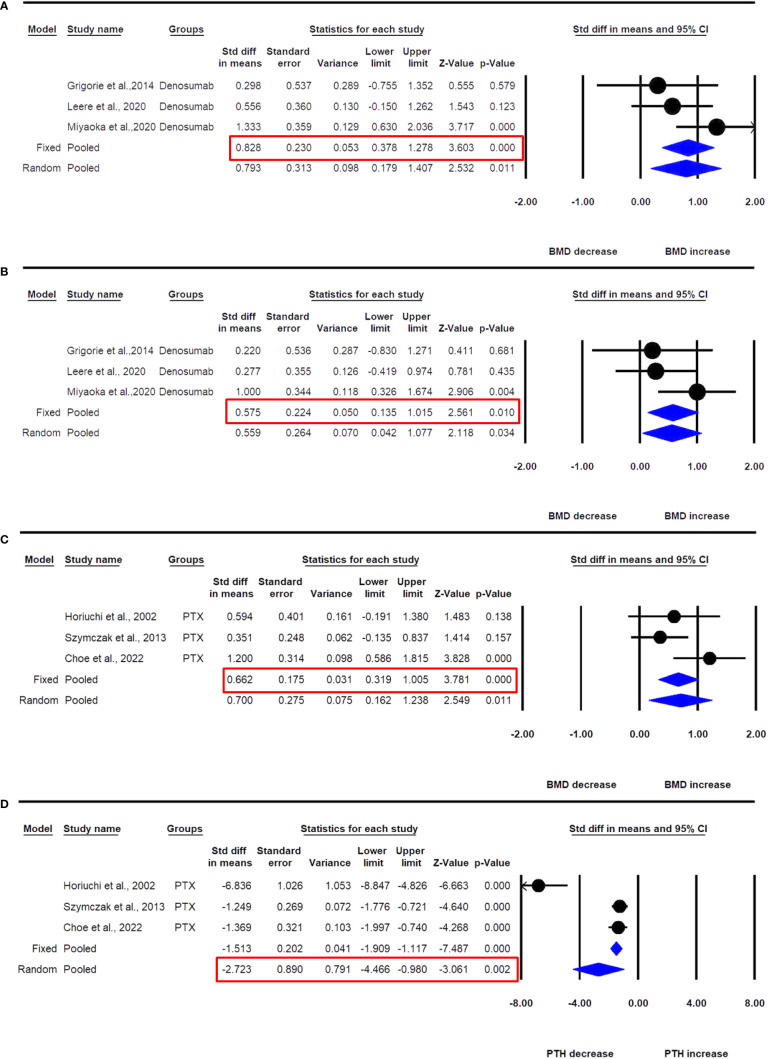
The effect of denosumab use in PHPT patients compared with baseline; **(A)** lumbar spine aBMD, **(B)** femur neck aBMD; and PTX on **(C)** lumbar spine aBMD, and **(D)** PTH in PHPT patients.

Three datasets from as many studies were available for lumbar spine aBMD before and 6- or 12 months after denosumab use. The fixed effect model was used for drawing inference because no significant heterogeneity was observed between these studies (I^2^=43.283, Q=3.526, p=0.172). Pooled analysis showed that the mean lumbar spine aBMD significantly increased after denosumab administration compared with the baseline (SDM=0.828, 95% CI=0.378 to 1.278, p=0.0001) (**Figure 6A**).

Three datasets from as many studies were available for femur neck aBMD before and 6- or 12 months after denosumab use. No significant heterogeneity was observed between these studies (I^2^=24.937, Q=2.664, p=0.264), suggesting the use of the fixed effect model for drawing a conclusion. Pooled analysis showed that the mean femoral neck aBMD significantly increased after denosumab administration compared with the baseline (SDM=0.575, 95% CI=0.135 to 1.015, p=0.010) (**Figure 6B**).

Only one study (31) had denosumab data for 24 months, and it was not in the required format, thus resulting in its exclusion. We could not analyze the biochemical parameters due to the limitation in data availability.

### 3.6 Effect of PTX on aBMD and serum PTH levels compared with baseline

In this meta-analysis, we focused on the effect of BPs and denosumab but not PTX in PHPT patients. We included only those studies where the data associated with PTX was provided as additional information for the selected studies.

Three datasets from as many studies were available for lumbar spine aBMD before and 12 months after PTX. There was no significant heterogeneity between these studies (I^2^=56.045, Q=4.550, p=0.103), suggesting the use of the fixed effect model for data analysis. In a pooled analysis, the mean lumbar spine aBMD significantly increased after PTX compared with baseline (SDM=0.662, 95% CI=0.319 to1.005, p=0.0001) (**Figure 6C**).

Three datasets from as many studies were available for serum PTH after 12 months of PTX. Significant heterogeneity was observed between these studies (I^2^=92.880, Q=28.090, p=0.0001), suggesting the use of the random effects model for drawing inference. In the pooled analysis, the mean serum PTH significantly decreased after PTX compared with baseline (SDM=-2.723, 95% CI=-4.466 to -0.980, p=0.002) (**Figure 6D**).

### 3.7 Effect of 24 months of ALN use on aBMD, PTH, and calcium compared with baseline

**Figure 7:**
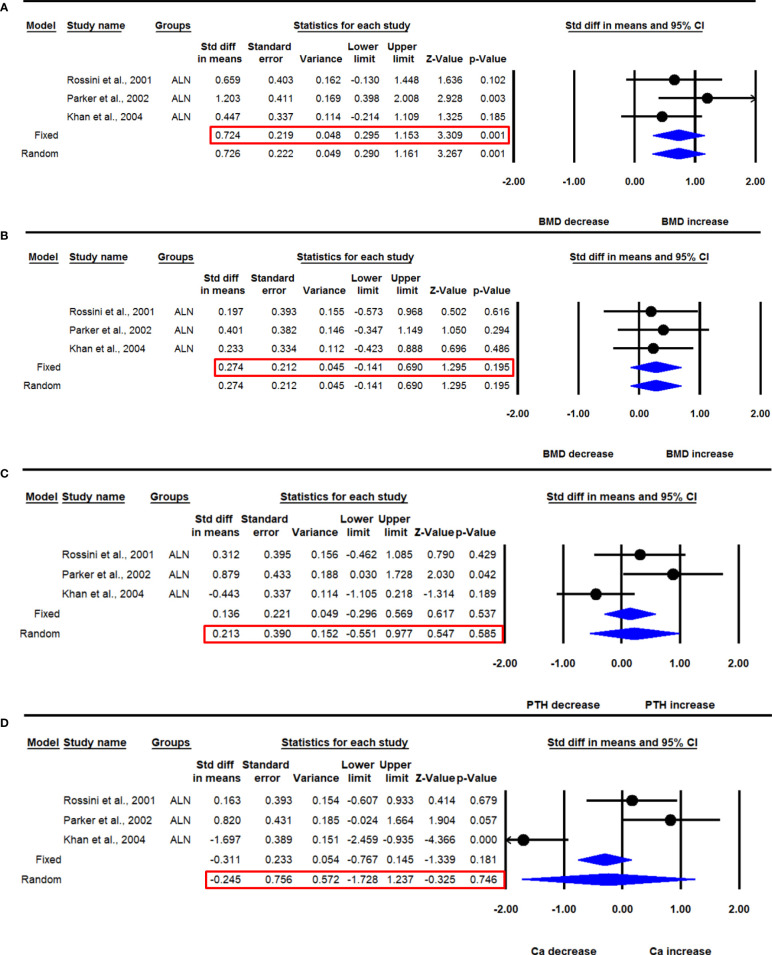
The effect of 24 months ALN administration on various parameters in PHPT patients compared with baseline; **(A)** lumbar spine aBMD, **(B)** femur neck aBMD, **(C)** serum PTH, and **(D)** serum calcium (Ca).

The majority of the data was available for 12 months, and 24 months of ALN administration from 3 studies. Three datasets from as many studies were available for lumbar aBMD before and 24 months after ALN use. The heterogeneity was not significant between these studies (I^2^=2.766, Q=2.057, p=0.358), suggesting the use of the fixed effect model for drawing a conclusion. Pooled analysis showed a significant increase in mean lumbar spine aBMD after ALN use (SDM=0.724, 95% CI=0.295 to 1.153, p=0.001) (**Figure 7A**).

Three datasets from as many studies were available for femoral neck aBMD before and 24 months after ALN use. The heterogeneity in these studies was not significant (I^2^=0.000, Q=0.164, p=0.922), suggesting the use of the fixed effect model for data analysis. Pooled analysis showed no significant change in the mean femoral neck aBMD after ALN administration (SDM=0.274, 95% CI=-0.141 to 0.690, p=0.195) (**Figure 7B**).

Three datasets from as many studies were available for serum PTH before and 24 months after ALN use. The heterogeneity among these studies was significant (I^2^=67.163, Q=6.091, p=0.048), suggesting the use of the random effects model for drawing inference. Pooled analysis showed that the mean PTH was not significantly changed after ALN administration (SDM=0.213, 95% CI=-0.551 to 0.977, p=0.585) (**Figure 7C**). Of the three studies, one (18) was sensitive; however, meta-analysis excluding this could not be performed due to the paucity of the number of studies.

Three datasets from as many studies were available for serum calcium before and 24-months after ALN use. The heterogeneity was significant between these studies (I^2^=90.505, Q=21.064, p=0.0001), suggesting the use of the random effects model for drawing conclusion. Pooled analysis showed a significant decrease in serum calcium after ALN administration (SDM=-0.245, 95% CI=-1.728 to 1.237, p=0.746) (**Figure 7D**).

### 3.8 Publication bias

The majority of the parameters were unaffected by publication bias. The unbiased estimates based on the trim and fill procedure have been mentioned where they have been observed.

### 3.9 Sensitivity analysis

The sensitivity analysis was performed with the exclusion of one study at a time. No study was found to be sensitive.

## 4 Discussion

A recent paper performed meta-analyses of all available medical and surgical modalities for PHPT in comparison to placebo control and excluded non-RCT studies where endpoint effects were compared with baseline (33). The parameters included in the meta-analysis were BMD, PTH, and calcium, although for BMD, only a single dataset was used for performing the meta-analysis. Our study examined whether anti-resorptive medicines may improve BMD in PHPT patients, and we focused on BPs and denosumab because there were sufficient studies on these medications to do meta-analysis. The parameters included in our study were BMD, calcium, phosphate, PTH, and BTMs. We included studies having placebo control as well as comparison between endpoints and baseline, i.e., both RCT and non-RCT. This way, we could most comprehensively capture the effects of anti-resorptives on bone and mineral homeostasis in order to determine their efficacy in protecting those PHPT patients from osteoporotic fractures who are ineligible for surgery.

We found aBMD gains in nine and three studies with BPs (7 ALN, 1 etidronate, 1 neridronate) and denosumab for 12 months, respectively; three with ALN and one with denosumab for 24 months, respectively. In the pooled analysis, BPs and denosumab use in PHPT patients for 12 months increased aBMD in the lumbar spine and femoral neck while decreasing serum calcium and phosphate. When the effects of only BPs were considered at this treatment duration, significant increases in lumbar spine aBMD, decreases in BTMs (OCN, BALP, and CTX-I), and decreases in serum calcium were observed. Of the BPs, sufficient studies were available only with ALN to conduct a meta-analysis, which revealed that ALN increased aBMD at the lumbar spine at 12- and 24 months but it did not affect aBMD at the femoral neck or the distal radius. ALN also lowered serum calcium while increasing serum PTH, but PTX normalized the hormone after 12 months. Serum PTH increased significantly after 12 months but returned to baseline 24 months after ALN use. Serum calcium levels dropped significantly after 12 months and returned to baseline after 24 months of ALN therapy. Regarding the skeletal effect, denosumab was superior to ALN and PTX as it increased aBMD at the lumbar spine as well as at the femoral neck.

PTX is the standard of care for symptomatic PHPT as well as in selected patients of asymptomatic PHPT. However, PTX can result in uncontrolled bone mineralization and hypocalcemia, a condition known as “hungry bone syndrome (HBS)” (34). A significant number of PHPT patients are unable to undergo PTX (asymptomatic, personal wish, severe comorbidities, and advanced age) (35). Mitigating hypercalcemia and its consequences, including improving BMD and reducing fracture risk, are priorities in managing such cases. Medical management for PHPT is not new. Anti-resorptives have been used to prevent bone loss, reduce the risk of fracture, and correct hypercalcemia although often temporarily. Cinacalcet has been used to decrease PTH secretion and hypercalcemia. Mithramycin has been historically used to normalize calcium (36). The main purpose of anti-resorptive therapy is to provide functional remission, i.e., normalizing calcium and preventing BMD loss.

Our meta-analysis findings support the long-term use of ALN and denosumab in providing skeletal protection in PHPT patients who are ineligible for surgery or in cases of surgery delay. ALN raised aBMD at the lumbar spine as compared to placebo (**Figure 5A**), while denosumab increased both aBMD at the lumbar spine and femoral neck when compared with baseline (**Figures 6A, B**). The effect on aBMD was accompanied by a reduction in BTM parameters, including serum calcium, OCN, BALP, and CTX-I. ALN had similar effects on BMD improvement and BTM reduction in men and women with PHPT (27). ALN at 12 months decreased serum calcium; however, the effect disappeared at 24 months. The effect of ALN in lowering serum calcium is inconsistent due to study heterogeneity and insufficient number of studies that spanned for 24 months. There was no effect on serum phosphate with ALN use. Only two denosumab studies measured serum calcium; in one, a decline in serum calcium was observed during the first month, after which it returned to baseline levels and continued until 50 weeks (21); and in the second, a decrease in serum calcium was observed in the first two weeks but then returned to the baseline levels and continued throughout the study (6 months) (26). PHPT is characterized by cortical bone loss with relative preservation of trabecular bone. In our meta-analysis, we observed that the increase in aBMD was greater at the trabecular site of the lumbar spine than the cortical site of the femur neck with both ALN and denosumab. From these results, it appears that the use of anti-resorptives in PHPT may have an impact similar to postmenopausal osteoporosis in terms of slowing bone remodeling but may fall short of significant reductions in the modeling (at the cortical bone) and continuous BMU activation at the endosteal surface as a consequence of increased PTH levels.

BMD is an important predictor of fracture risk, but fracture data is essential to determine the treatment efficacy in osteoporosis. We found that only two studies addressed fractures, and both observed no significant effect of BPs on the rate of fragility fracture (30) and fracture risk (29) in PHPT patients. Given insufficient data, we could not perform a meta-analysis of the effect of BPs in modifying the risk of fracture and hence propose future studies to acquire fracture data.

Strong suppression of bone resorption by anti-resorptive therapy in PHPT could lead to the exacerbation of hyperparathyroidism. By a pooled analysis, we observed there was an increase in serum PTH following 12 months of ALN use. In the case of denosumab, one study reported a moderate yet significant rise in PTH after 12 months (25); while in another study conducted for 50 weeks, a rapid increase in PTH level over the baseline was quickly followed by its return to the baseline level till the end of the study (21). Unlike ALN, pooled analysis of the effect of denosumab on PTH levels after 12 months could not be done due to the paucity of data. Only one study examined the short-term effect of denosumab (3 and 6 months) on PTH levels in PHPT patients and found that the drug had no effect (26). Future studies are required to assess the effect of denosumab on PTH levels in PHPT patients. These studies are essential for assessing the safety of long term denosumab in PHPT as elevation of PTH has been reported to be associated with adverse effects, including hypertension, left ventricular hypertrophy, heart failure, and renal insufficiency (37).

Furthermore, the production of fibroblast growth factor 23 (FGF23) is increased by PTH, and the former is an independent marker for left ventricular function (38). Thus, further elevation of serum PTH using BPs in PHPT could heighten cardiovascular risk. Future studies should measure FGF23 levels and monitor for any cardiovascular event in PHPT patients treated with anti-resorptive drugs.

Theoretically, a calcimimetic drug that inhibits both PTH and FGF23 can be combined with anti-resorptives for greater efficacy and preventing cardiovascular morbidity. A clinical trial has considered this combination and found that cinacalcet improved the biochemical abnormalities and alendronate increased BMD at 24-months follow-up (35). However, more such studies are required to determine the efficacy and safety of these combinations through a meta-analysis.

The strengths of this meta-analysis are that a comparison of drug effects has been made with both placebo and baseline, and drug effects at the site-specific BMD and BTMs have been compared with PTX. The limitations include the inclusion of both RCT and non-RCT studies and the lack of fracture data due to insufficient data availability. Head-to-head comparison between anti-resorptive and PTX therapy is not possible because of the paucity of the studies.

## 5 Conclusion

Anti-resorptive therapies, including ALN and denosumab, increase aBMD, decrease serum calcium, and inhibit BTMs in PHPT patients. Alendronate significantly increases PTH levels in PHPT patients compared with both baseline and placebo without affecting normal mineral levels. Future studies should measure FGF23 and monitor cardiovascular events in PHPT patients receiving anti-resorptive drugs. A combination of calcimimetic and anti-resorptive drugs could provide an improved clinical profile over monotherapy to treat aberrant bone and mineral homeostasis in PHPT patients.

## Data availability statement

The original contributions presented in the study are included in the article/**Supplementary Material**. Further inquiries can be directed to the corresponding authors.

## Author contributions

SwR conducted literature screening, statistical analyses of the extracted data and wrote the manuscript; AD performed statistical analyses of the extracted data and wrote the manuscript; SiR, AM, and NC conceived the idea, conducted literature screening, and finalized the manuscript. All authors contributed to the article and approved the submitted version.
