# Supplementary material for: Efficacy of antiresorptive agents bisphosphonates and denosumab in mitigating hypercalcemia and bone loss in primary hyperparathyroidism: A systematic review and meta-analysis
Source: Front Endocrinol (Lausanne). 2023 Feb 2;14:1098841. doi: 10.3389/fendo.2023.1098841 (PMC9931892; doi:10.3389/fendo.2023.1098841)
Supplement: Supplementary file 1 [file DataSheet_1.docx]

**Supplementary Material**

**Efficacy of antiresorptive agents bisphosphonates and denosumab in mitigating hypercalcemia and bone loss in primary hyperparathyroidism: a systematic review and meta-analysis**

Swati Rajput^1,2^, Aditya Dutta^3^, Singh Rajender^1^, Ambrish Mithal^3,*^, Naibedya Chattopadhyay^1,2*^

1. Division of Endocrinology and Centre for Research in Anabolic Skeletal Targets in Health and Illness (ASTHI), CSIR-Central Drug Research Institute, Sector 10, Jankipuram Extension, Sitapur Road, Lucknow 226031, India.
2. Academy of Scientific and Innovative Research (AcSIR), Ghaziabad- 201002, India.
3. Division of Endocrinology and Diabetes, Medanta-The Medicity Hospital, Haryana, India

*Correspondence: A. Mithal, [ambrishmithal@hotmail.com](mailto:ambrishmithal@hotmail.com) or N. Chattopadhyay, [n_chattopadhyay@cdri.res.in](mailto:n_chattopadhyay@cdri.res.in)

**Disclosures:** Swati Rajput, Aditya Dutta, Singh Rajender, Ambrish Mithal, and Naibedya Chattopadhyay declare that they have no conflict of interest.

**Ethics approval:** Not applicable.

**Consent to participate:** Not applicable.

**Consent for publication:** Not applicable.

**Availability of data and material:** All data are available upon request.

**Author’s contributions:** SR conducted literature screening, statistical analyses of the extracted data and wrote the manuscript; AD performed statistical analyses of the extracted data and wrote the manuscript; SR, AM, and NC conceived the idea, conducted literature screening, and finalized the manuscript.


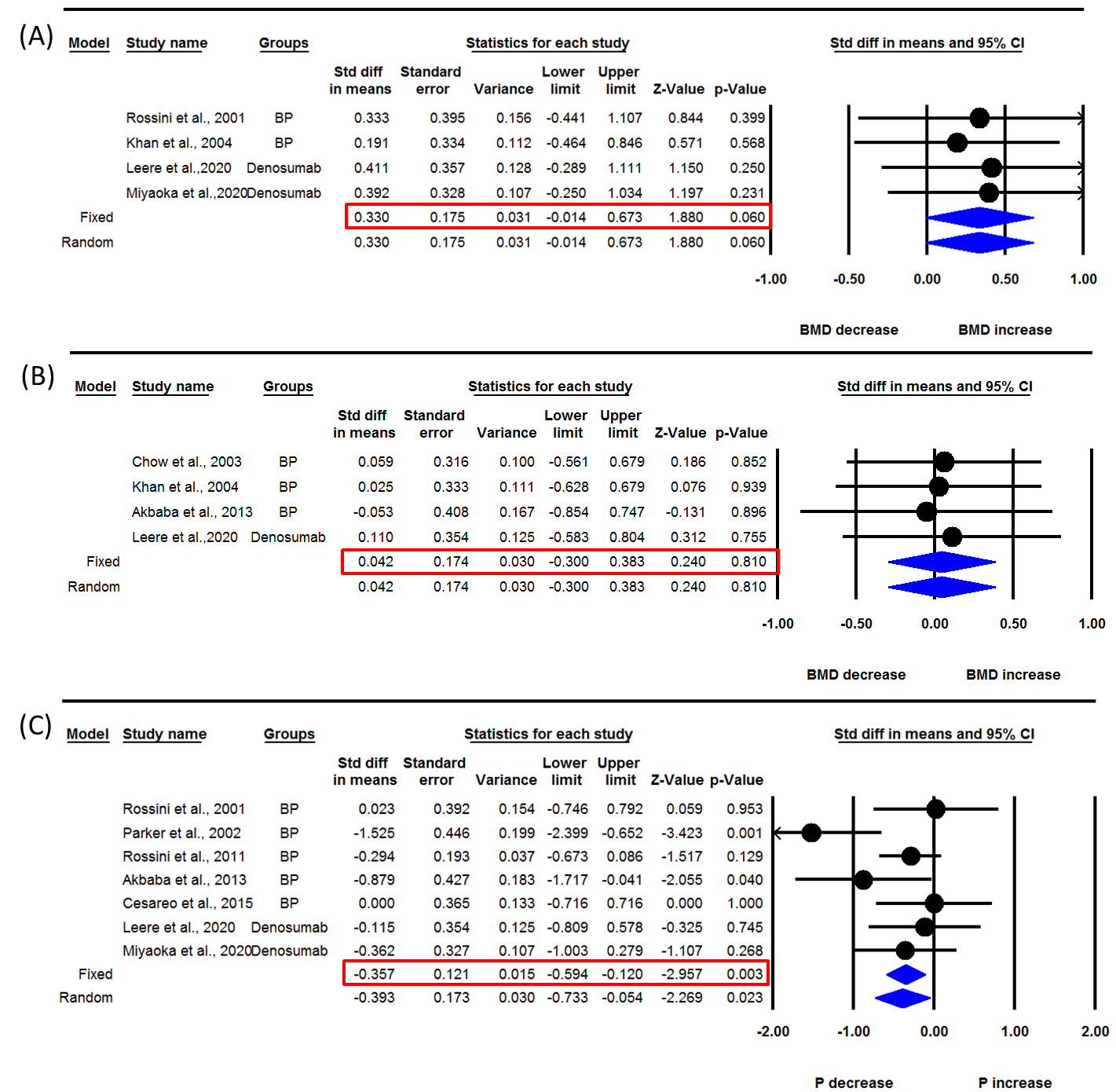


Figure 1: Effect of drug treatment on various parameters in PHPT patient compared with baseline; (A) total hip aBMD, (B) distal radius aBMD, and (C) serum phosphate (P).


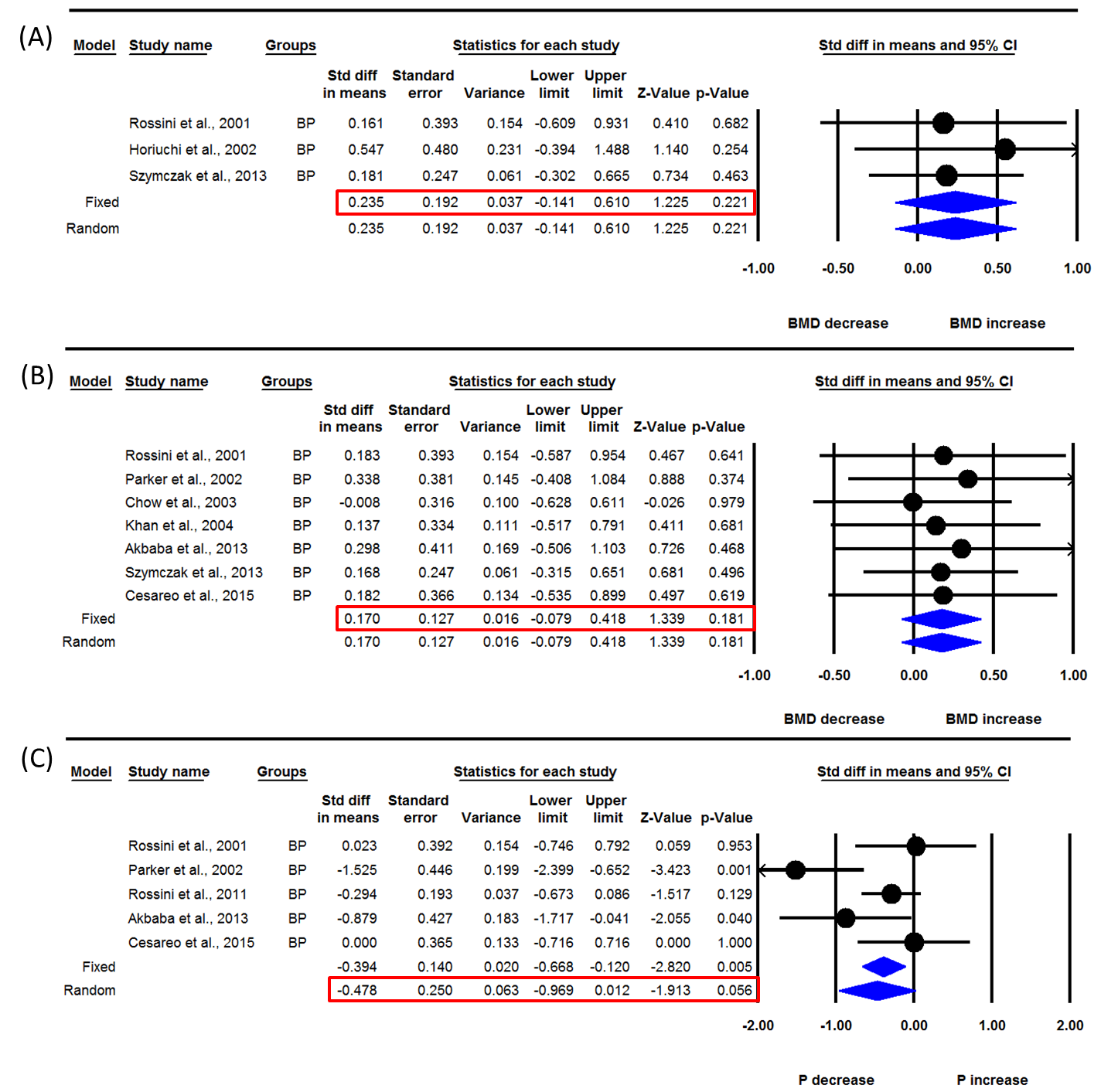


Figure 2: Effect of BP treatment on various parameters in PHPT patients compared with baseline; (A) total aBMD, (B) femur neck aBMD, and (C) serum phosphate (P).


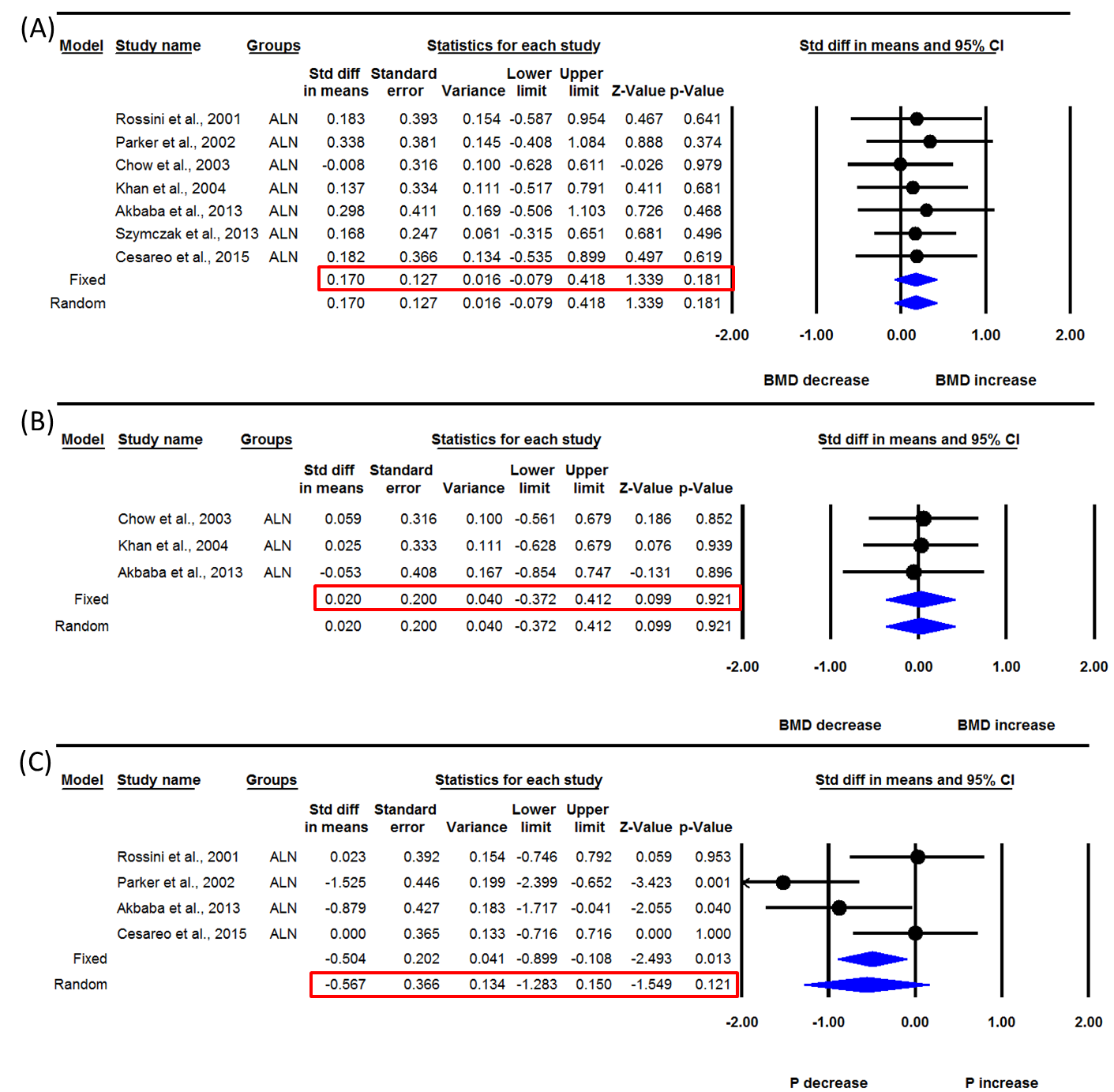


Figure 3: Effect of ALN treatment on various parameters in PHPT patients compared with baseline; (A) femur neck aBMD, (B) distal radius aBMD, and (C) serum phosphate (P).


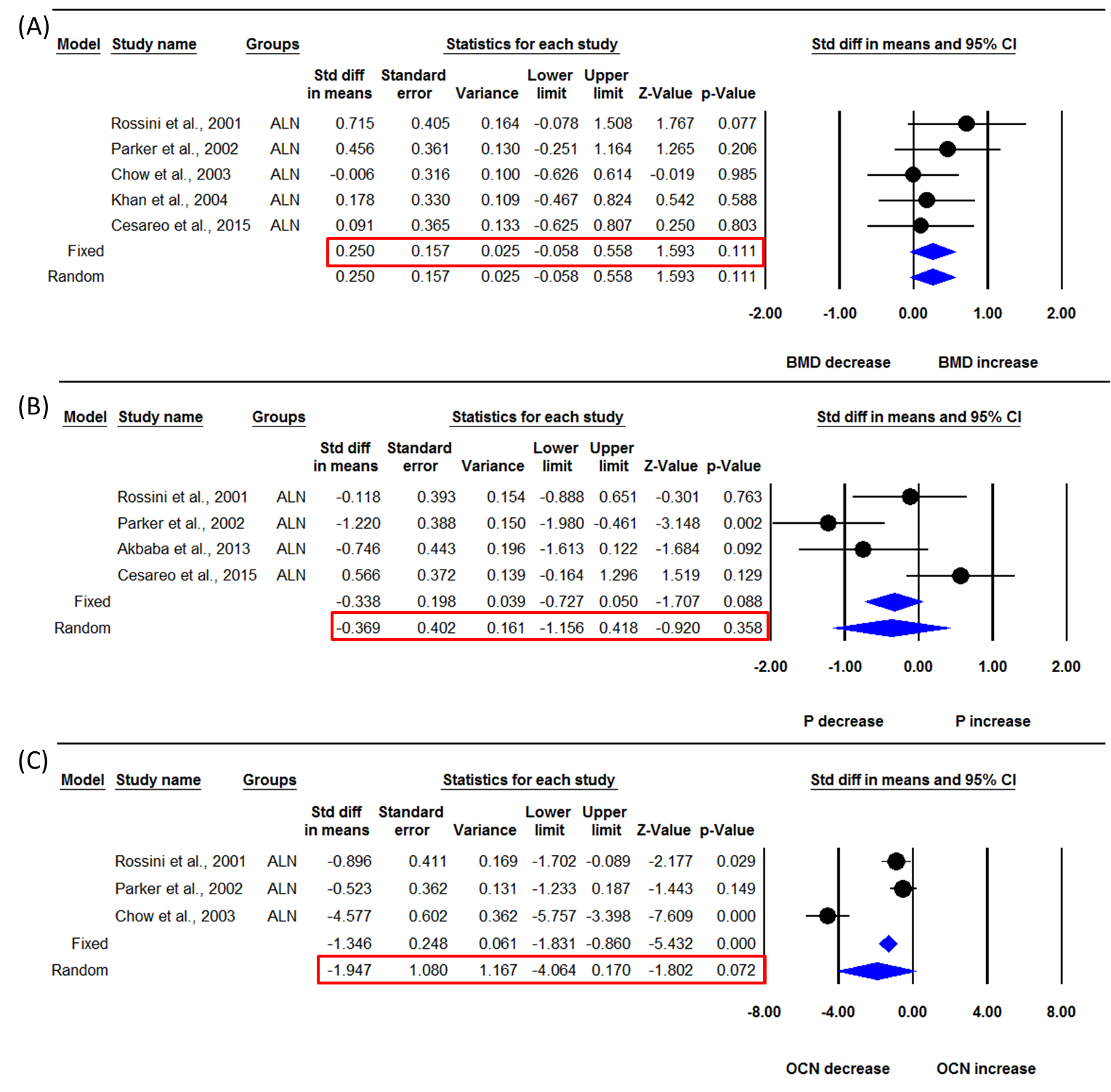


Figure 4: Effect of ALN treatment on different parameters in PHPT patients compared with placebo control; (A) femoral neck aBMD, (B) serum phosphate (P), and (C) serum osteocalcin (OCN).
